# A New Proton Transfer Complex Between 3,4-Diaminopyridine Drug and 2,6-Dichloro-4-nitrophenol: Synthesis, Spectroscopic Characterization, DFT Studies, DNA Binding Analysis, and Antitumor Activity

**DOI:** 10.3390/molecules29215120

**Published:** 2024-10-30

**Authors:** Reem M. Alghanmi, Maram T. Basha, Ahlam I. Al-Sulami, Saied M. Soliman, Laila H. Abdel-Rahman

**Affiliations:** 1 Department of Chemistry, College of Science, University of Jeddah, P.O. Box 80327, Jeddah 21589, Saudi Arabia; mtbasha@uj.edu.sa (M.T.B.); aialsulami@uj.edu.sa (A.I.A.-S.); 2 Department of Chemistry, Faculty of Science, Alexandria University, P.O. Box 426, Ibrahimia, Alexandria 21525, Egypt; saied1soliman@yahoo.com; 3 Chemistry Department, Faculty of Science, Sohag University, Sohag 82534, Egypt; laila.abdelrahman@science.sohag.edu.eg

**Keywords:** proton transfer, 3,4-diaminopyridine, nitrophenol, optimized geometry, HOMO and LUMO, MEP, TD-DFT, DNA binding, cytotoxicity

## Abstract

The proton transfer (PT) complexation reaction between 3,4-diaminopyridine (3,4-DAP), an important drug, and 2,6-dichloro-4-nitrphenole (DCNP) was investigated experimentally and theoretically. The experimental results indicated a chemical reaction occurred because of a hydrogen bonding, followed by proton transfer from the DCNP to the 3,4-DAP in different polar media. The Benesi–Hildebrand equation was used to estimate the formation constant (*K*_f_), molar absorptivity (ε_PT_), and other physical parameters. The formed PT complex was characterized using FTIR, ^1^H, and ^13^C NMR spectra. In addition, the nanocrystalline structure, particle sizes, and surface morphology of the complex were investigated by XRD and SEM-EDX. The structure of the 1:1 PT complex was calculated theoretically in the gas phase and the presence of solvent effects. Using TD-DFT calculations, the band observed at 406 nm (Calc. 379.5 nm) and 275 nm (Calc. 272.3 nm) could be assigned to the HOMO→LUMO transition (99%), and HOMO→L+3 transition (87%), respectively. The DNA binding ability of the PT complex was investigated, revealing an intercalative binding mechanism with a binding constant K_b_ of 4.6 × 10^4^ M^−1^. Based on the results of the Ct-DNA binding study, the binding free energy of the PT complex with the receptor of human DNA (PDB ID:1BNA) is found to be −7.2 kcal/mol. The cytotoxic effects of the PT complex were evaluated on selected cancer cell lines, demonstrating significant antitumor activity against A-549 and MCF-7 cancer cell lines.

## 1. Introduction

Investigation of the chemistry of drug interaction with selected organic molecules, whether through charge transfer (CT), proton transfer (PT), or hydrogen bonding (HB), has received increasing attention in recent decades because of their significance in various energy transfer processes such as electrochemical and biochemical processes [1,2,3,4]. These interactions could form stable complexes, potentially useful for drug vectorization or developing drug combinations with enhanced properties. Physicochemical properties and weak interactions are potential links between drugs and their biological properties. However, finding suitable receptors is challenging. Proton donors or electron-acceptors are used to study these physicochemical properties responsible for CT and PT complexes of drugs [5]. Studying the physicochemical properties of these complexes also provides essential data about the mechanism of pharmacological drug activity [6]. Furthermore, CT, PT, and HB complexes are well known for their different biological activities, such as anticancer, antitumor, and antimicrobial activities [7,8,9,10].

In addition, investigating DNA binding with CT or PT complexes is crucial in biological research, where DNA is the primary target for developing novel antiproliferative drugs and anticancer therapies [11,12,13,14]. UV−visible spectroscopy is one of the most used methods to investigate the binding mechanism between the CT and PT complexes and DNA. The interaction of DNA is essential for comprehending the molecular mechanism underlying the activity of CT and PT complexes. Thus, understanding the binding mechanisms of these minuscule molecules to DNA may facilitate the advancement of novel pharmaceuticals, diagnostic probes, and reactive agents [15]. Therefore, investigating CT, PT, and HB interactions is crucial for chemistry, biology, and pharmacology.

The hydrogen bond (HB) is a weak interaction between molecules and/or ions in which a hydrogen atom (H) from a donor molecule (D) forms a connection with an acceptor molecule (A) through the hydrogen atom. HB of type A**...**H-D ↔ AH^+^**...**D^−^ is characterized by a proton transfer equilibrium inside these bonds. The PT equilibrium can be investigated via X-ray diffraction (XRD), UV-Vis, FTIR, and NMR spectroscopic methods. Phenol and its derivatives, such as nitrophenols, form PT complexes with oxygen and nitrogen bases, making it one of the most extensively studied hydrogen-bonded complexes. The hydrogen-bonded complexes (A**...**H-D) and ion pair (AH^+^**...**D^−^) in nitrophenol are relatively easy to distinguish in the UV-vis spectra. This is due to the fact that the ^1^L_b_ transition of nitrophenol is approximately 300 nm, whereas that of nitrophenolate ions is approximately 400 nm [16]. The PT complexes of nitrophenols with various heterocyclic bases, such as piperidines, pyridines, and pyrimidines, have been extensively studied as H-bonded systems in the solution, solid, and gas phases [12,17,18,19,20,21,22,23].

**Scheme 1:**
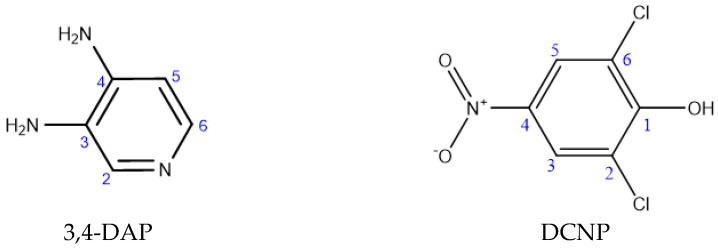
The chemical structure of 3,4-DAP and DCNP with carbon numbering.

Aminopyridines are a significant group of compounds that may either donate electrons or accept protons. By studying their PT complexes, we can obtain insight into several chemical reactions in which they participate. Studies have demonstrated that they possess a range of pharmacological and medicinal uses alongside their use in analytical chemistry [15,24,25]. The molecule 3,4-DAP is a pyridine derivative that creates CT complexes with acceptors [3,4]. This molecule has two sites for donation: the nitrogen atom in the pyridine ring and the two amino groups. The 3,4-DAP molecule is a powerful heterocyclic chemical that selectively blocks the potassium channels of squid axon membranes, and it has been used in patients with Lambert–Eaton myasthenic syndrome (LEMS) [26,27]. It has been documented that 3,4-DAP has advantageous effects in individuals diagnosed with specific congenital myasthenic syndromes (CMSs) [28,29]. The 3,4-DAP molecule has several reactive sites, including pyridine nitrogen atom and amino groups at positions 3 and 4, as shown in Scheme 1, which could react as an electron donor or proton acceptor.

Based on the biological and pharmaceutical importance of 3,4-DAP, along with our ongoing interest in investigating its proton transfer (PT) and charge transfer (CT) interactions [7,12,23,26,27,28,29], it is noteworthy that limited information is currently available on the PT complexes of 3,4-DAP. Our previous work confirmed that 3,4-DAP is a promising electron donor molecule with a high propensity to form hydrogen bonds (as a hydrogen acceptor) [3,4]. So, the current study focuses on the investigation of the PT reaction in the amine-phenol system between 3,4-DAP as a proton acceptor and 2,6-dichloro-4-nitrophenol (DCNP), which is one of the most acidic nitrophenols (p*K*_a_ = 3.55) [30], as a proton donor. Electronic absorption data on the PT reaction between 3,4-DAP as the proton acceptor and DCNP as the proton donor in EtOH and MeCN were provided. The molecular composition, formation constants, and spectroscopic physical properties of the new PT complex were calculated and evaluated. In addition, the elemental composition and characterization of the synthesized solid complex was determined using UV–vis, FT-IR, ^1^H and ^13^C NMR, XRD, and SEM-EDX spectra. The experimental results were confirmed through DFT computational analysis. Furthermore, A-549 cells represent a widely studied model for non-small cell lung cancer, reflecting the pathophysiology of lung tumors and their response to various treatments. MCF-7 cells, on the other hand, are a well-established model for estrogen receptor-positive breast cancer, allowing for the investigation of hormonal influences on tumor growth and drug sensitivity. Both cell lines provide a robust platform to assess the anticancer potential of the PT complex, as they are responsive to DNA-interactive compounds. By determining the IC50 values, this study aims to elucidate the cytotoxic mechanisms of the PT complex and its interaction with cellular DNA, contributing to the development of innovative therapeutic strategies in cancer treatment.

## 2. Results and Discussion

### 2.1. Solution State Studies

#### 2.1.1. Observation of the PT Complex Band

**Figure 1:**
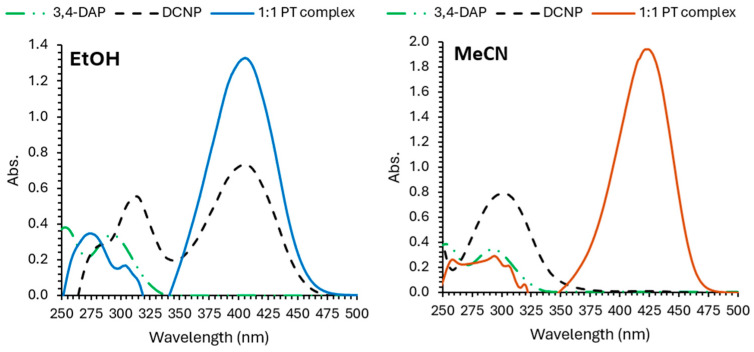
Electronic absorption spectra of 3,4-DAP; DCNP and PT complex in EtOH, and MeCN at room temperature.

When nitrophenol derivatives such as DCNP (D) form an H-bonded molecular complex (A**...**H-D) with H-acceptor (A), the UV-vis spectrum arising from the electronic transitions exhibits perturbations in both intensity and frequency. However, these alterations often remain moderate compared with free nitrophenol [16,31]. The transformation of nitrophenol into its corresponding nitrophenolate leads to a shift towards longer wavelengths (redshift) of the ^1^L_a_ and ^1^L_b_ absorption bands, accompanied by an increase in ε*_max_* [32]. The ^1^L_b_ transition of nitrophenols occurs at around 300 nm, whereas that of nitrophenolate occurs at approximately 400 nm. When PT ion pairs (AH^+^…D) are formed, the electronic spectrum is similar to that of nitrophenolate ions [16,31]. The UV-vis spectra of DCNP, 3,4-DAP, and their mixture in EtOH are shown in Figure 1, where two bands at 313 and 405 nm are present in the DCNP spectrum. The band at 405 nm has a relatively low intensity and indicates the incomplete dissociation of DCNP into its ion [33]. The complex spectrum showed that the intensity complex of the band at λ_max_ 405 nm is higher than that of the free DCNP band, and the solution color changed from extremely pale yellow to brilliant yellow as a result, Figure S1 (Supplementary Materials). This observation indicates the formation of the PT complex and shifts equilibrium toward the formation of the H-bonded ion pair [(3,4-DAP)^+^(DCNP)^−^].

On the other hand, the DCNP spectrum in MeCN has one band appearing at λ_max_ 305 nm, as shown in Figure 1. A bright yellow solution formed after mixing the DCNP solution with the 3,4-DAP solution, associated with a redshift in the phenol band. The new band was observed at λ_max_ 423 nm, corresponding to phenolate moiety in solution, suggesting a more significant separation of molecules in the PT complex to form the H-bonded ion pair [(3,4-DAP)^+^(DCNP)^−^]. To ensure that the complex and DCNP bands were not overlapping, the spectra of the complex was recorded against a solution of DCNPs at the same concentration.

#### 2.1.2. Molecular Composition of the PT Complex and Its Stability

**Figure 2:**
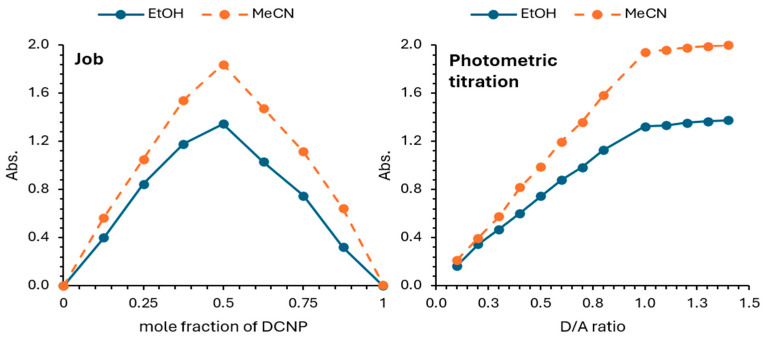
Job’s method of continuous variations and spectrophotometric titration plots of PT complex in different solvents.

The molecular composition of the PT complex was determined in EtOH and MeCN at λ_max_ using different spectrophotometric methods. Figure 2 shows the plots of Job’s method of continuous variations and spectrophotometric titration method. Both methods indicated the formation of a 1:1 PT ion pair, [(3,4-DAP)^+^(DCNP)^−^], indicating that the molecular composition of the PT complex is unaffected by the polarity of the solvent.

The stability of the formed complex was also studied by measuring the intensity of [(3,4-DAP)^+^(DCNP)^−^] bands in both solvents at various times. Figure S2 shows that the [(3,4-DAP)^+^(DCNP)^−^] was stable for more than 2 h in both solvents.

#### 2.1.3. Determination of Formation Constant and Some Spectroscopic Parameters

Figures S3 and S4 show the electronic spectra of the PT complex using various concentrations of 3,4-DAP (from 1.0 × 10^−5^ mol L^−1^ to 1.0 × 10^−4^ mol L^−1^) with a constant concentration of DCNP (1.0 × 10^−4^ mol L^−1^) in EtOH and MeCN, respectively. The absorbance of the complex was noticed to increase with an increase in the proton acceptor concentration (3,4-DAP). As the concentration of 3,4-DAP rose, the absorbance of the complex increased. Based on the absorbance values of the [(3,4-DAP)^+^(DCNP)^−^] complex at various concentrations of 3,4-DAP (Figures S3 and S4), the modified Benesi–Hildebrand method [34] (Equation (1)) was applied to determine the formation constant K*_f_* (L mol^−1^) and the molar absorptivity ε of the PT complex in EtOH and MeCN at room temperature (20 °C).

(1) $$\frac{\left[C_{D}\right]}{Abs}=\frac{1}{K_{f}\cdot \varepsilon }\cdot \frac{1}{\left[C_{A}\right]}+\frac{1}{\varepsilon }$$

**Figure 3:**
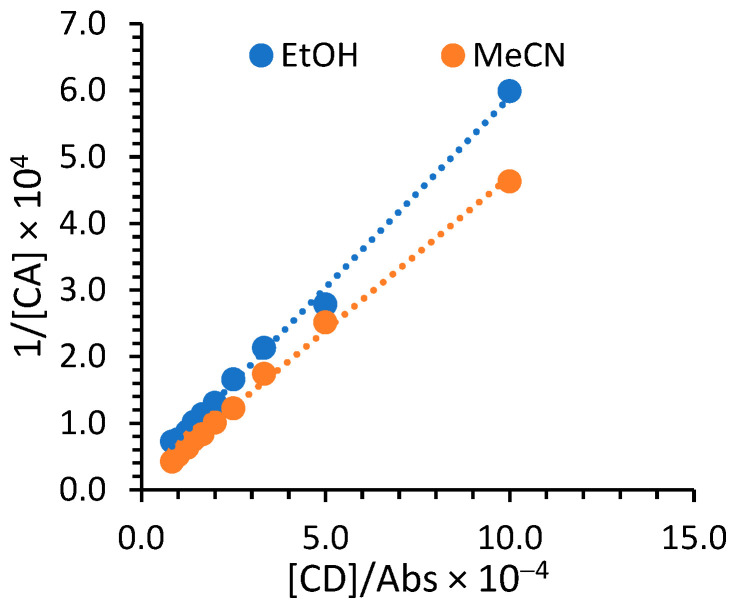
Bensie–Hildebrand plots of the PT complex in different solvents.

Straight lines were obtained by plotting [*C_D_*]/*Abs* versus 1/[*C_A_*] for the complex in EtOH and MeCN (Figure 3), where [*C_D_*] is the DCNP concentration, [*C_A_*] is the 3,4-DAP concentration, and *Abs* is the [(3,4-DAP)^+^(DCNP)^−^] absorbance at λ_max_. The K*_f_* and ε values are determined from the slope and intercept values of the lines, where the slope and intercept are equal to 1/K*_f_*ε and 1/ε, respectively. The results in both solvents are collected in Table 1. The K*_f_* and ε values for the [(3,4-DAP)^+^(DCNP)^−^] complex recorded high values, indicating its high stability in both solvents. The high stability was expected based on the high acidity of DCNP (p*K*_a_ = 3.55) [30] and the high basicity of the cyclic pyridine base 3,4-DAP (p*K*_a_ = 9.25) [35]. The highest value of K*_f_* was recorded in the EtOH system compared with the MeCN system. This finding could be explained according to Kamlet–Taft solvent parameters [36], α (hydrogen bond donor), β (hydrogen bond acceptor), and ∏* (dipolarity/polarizability). EtOH has near values of α (0.83) and β (0.77) and a relatively low value of ∏* (0.54), which causes a H-bond formation between EtOH and both 3,4-DAP and DCNP moieties, stabilizing the ground state of the complex, and raising K*_f_* value. In contrast, the smallest value of K*_f_* in MeCN could be explained through its high value of ∏* (0.75), which leads to the form of a dimer of MeCN molecules by self-association. This dimer increases steric hindrance and decreases the effective dipole moment, thus limiting the PT process and reducing the *K_f_* value to half that of EtOH, as shown in Table 1.

The value of K*_f_* was used to determine the standard free energy (ΔG°; kJ mol^−1^) using Equation (2) [37], while the value of ε*_max_* was used to determine the dipole moment *μ* [38] and the oscillator strength *f* [39], using Equations (3) and (4), and their values give information about PT interactions for the complex in both solvents.

(2) $$∆G{}^{\circ}=-2.303logK_{f}$$

(3) μ=0.0958εmax. Δν12/νmax

(4) f=4.32×10−9[εmax. Δν12]

where Δν_½_ and ν*_max_* are the half bandwidth and the maximum wavenumber of the PT band in cm^−1^, respectively, and ε*_max_* is the extinction coefficient of the PT band. The values of ΔG°, *f*, and *μ* are listed in Table 1. The negative values of ΔG° for the complex in both solvents indicate the presence of spontaneous interaction between 3,4-DAP and DCNP. Furthermore, the ΔG° in EtOH is larger than ΔG° in MeCN, which aligns with the corresponding findings for K*_f_*.

### 2.2. Solid State Studies

#### 2.2.1. Observation of the Solid PT Complex and Energy Gap

Different solvents were tested to dissolve the isolated solid-state complex of 3,4-DAP, and the electronic spectra of the dissolved complex were recorded. The tested solvents were EtOH, MeOH, MeCN, AcN, DMF, and CHCl_3_. DMF was found to be the best solvent for this purpose. The electronic molecular absorption spectrum of the new complex in DMF is shown in Figure S5. The new absorbance band at λ_max_ 430 nm, which differs from the reactants, suggests that the phenolate ion has formed.

**Figure 4:**
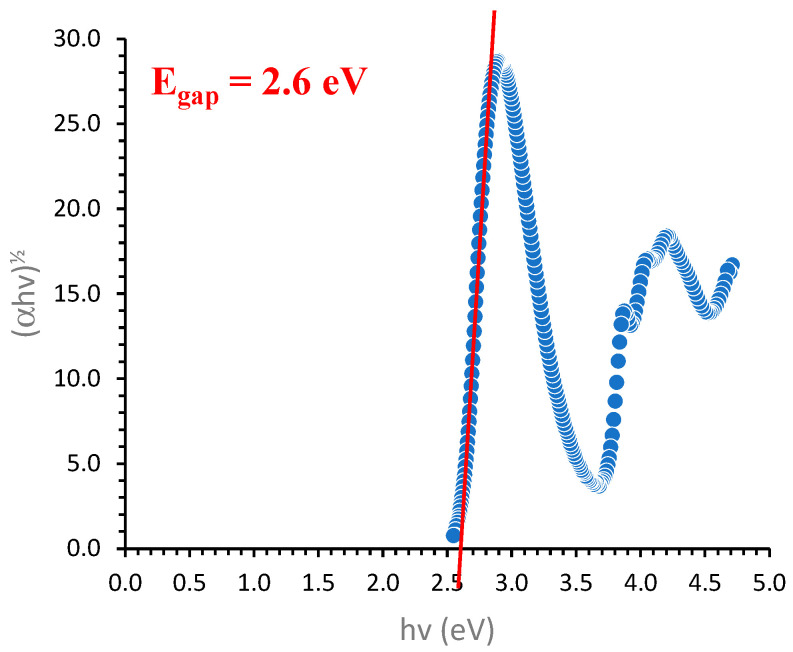
Tauc plot of the PT complex.

The energy gap, also known as the optical band gap (*E*g), is the lowest amount of energy needed for an electron to move from the valence band to the conduction band [40]. It may be calculated based on a transmittance of the complex (*T*). The optical absorption in the immediate region of the absorption band edge may be utilized to determine the energy of the bandgap (*E*g) and validate the synthesis of the PT complex. The absorption coefficient (*α*) is often calculated from the transmittance (*T*) of a complex using the following equation:

(5) $$\alpha =\frac{1}{d}lin\;\left(\frac{1}{T}\right)$$

where *d* is the width of the cell (*m*), the bandgap of the complex is then determined by the correlation between *α* and *E*g, as shown by the following equation [41]:

(6) $$\alpha h\nu =A{\left(h\nu -E_{g}\right)}^{m}$$

where *m* is ½ for direct transition, and *A* is an energy-independent constant. The values of (α*h*ν)^½^ were graphed versus *h*v at 20 °C to obtain a Tauc plot, as seen in Figure 4. The value of direct *E*_g_ was calculated by analyzing the linear portion of the Tauc plot at the absorption edge, where (α*h*ν)^½^ = 0 [40]. It was found that the *E*_g_ value is 2.60 eV, as shown in Figure 4, indicating the semiconducting behavior of the formed complex [42,43]. Lower values of *E*_g_ were obtained in the study of CT complexes of 3,4-DAP with CLA and DHBQ; their E_g_ values are 2.30 and 2.25 eV, respectively [3].

#### 2.2.2. FTIR Spectra

**Figure 5:**
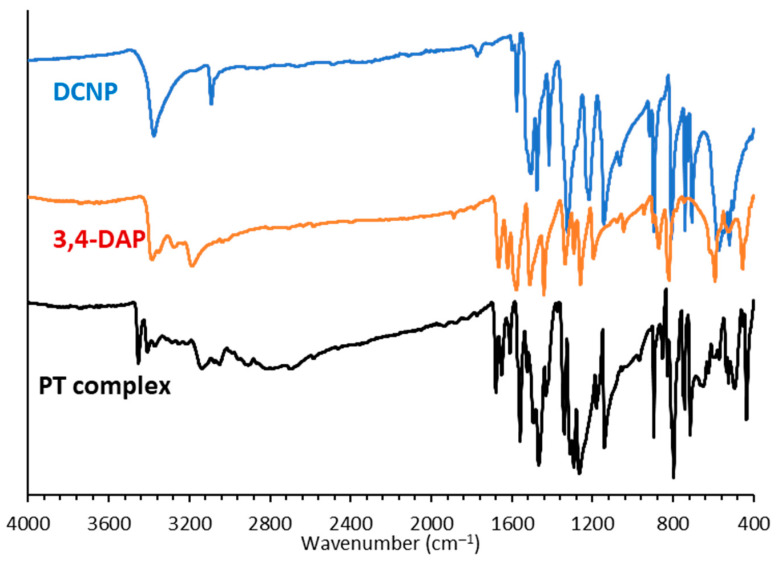
Infrared spectra of the free 3,4-DAP, free DCNP, and the PT complex in the range of 4000–400 cm^−1^.

The FTIR spectra of the individual components, namely free DCNP (acting as the proton donor), free 3,4-DAP (acting as the proton acceptor), and the 1:1 PT complex, are presented in Figure 5. All the FTIR bands observed for the reactants are mostly present in the spectrum of the complex, indicating the formation of the complex. On the other hand, the absorption peaks corresponding to DCNP and 3,4-DAP in the complex exhibit shifts in frequencies or changes in intensities, suggesting alterations in electron distribution due to complexation. These spectral alterations can be linked to the transfer of a proton from DCNP to 3,4-DAP. Notably, the disappearance of the ν(OH) band of free DCNP at 3376 cm^−1^ in the spectrum of the complex strongly supports the transfer of the phenolic proton to the nitrogen atom of the pyridine ring (Figure 5) [12,44]. Furthermore, the presence of the new broad absorption band at 2700 cm^−1^ in the complex spectrum can be ascribed to the participation of the pyridine nitrogen in the newly hydrogen-bonded PT complex (O^−^....HN^+^) [12,22,44].

In addition, the FTIR spectra reveal notable shifts in the stretching vibrations of the amino groups of 3,4-DAP upon complex formation. Specifically, the stretching vibration frequencies of both amino groups are shifted to higher frequencies of 3408 and 3373 cm^−1^ in the complex spectrum, compared with the spectrum of free 3,4-DAP, which appeared at 3386 and 3362 cm^−1^. Conversely, the stretching vibrations for the other amino group shift to lower frequencies at 3138 and 3055 cm^−1^, compared with 3298 and 3187 cm^−1^ in the spectrum of the 3,4-DAP. These shifts in amino group stretching vibrations suggest changes in the electronic environment around the amino groups, likely because of the formation of hydrogen bonds and the overall rearrangement of electron density in the complex. Moreover, it was observed the shifts in the frequencies of δ (NH_2_), ν (C=C), ν (C=N), and ν (C–N) in the complex spectrum at 1679, 1559, 1526, and 1340 cm^−1^, respectively, compared with 1667, 1578, 1512, and 1336 cm^−1^ of the free 3,4-DAP spectrum. These frequency shifts provide further evidence of complex formation, indicating alterations in the bonding environment and confirming the structural changes associated with proton transfer and complex stabilization.

Additionally, the shifts were observed in the asymmetric and symmetric stretching vibrations of the nitro group (ν^as^ (NO_2_) and ν^s^ (NO_2_)), supporting the formation of the studied complex. In the spectrum of free DCNP, these vibrations occur at 1577 and 1324 cm^−1^, respectively. Upon complex formation, they are shifted to 1610 and 1310 cm^−1^. Furthermore, the spectrum of the complex shows shifts in stretching vibrations ν (C–Cl) at 898 and 798 cm^−1^, as opposed to 896 and 809 cm^−1^ in the spectrum of free DCNP (Figure 5). The FTIR spectra, therefore, provide clear evidence that the nitrogen atom of the pyridine ring in the protonated 3,4-DAP participates in hydrogen bonding with the deprotonated phenolic group of DCNP. This interaction facilitates the newly formed 1:1 hydrogen-bonded PT complex, which is characterized by a significant redistribution of electron density and stabilization through hydrogen bonding interactions.

#### 2.2.3. NMR Spectra

Figures S6–S11 show the ^1^H and ^13^C NMR spectra of the investigated compounds in DMSO*-d*_6_, respectively. The ^1^H NMR spectra reveal the resonance signals of free reactants within the complex, indicating the complex formation. It was noticed from the spectrum of the studied complex that a downfield shift in most of the resonance signals indicated proton transfer from DCNP to 3,4-DAP. In addition, the signal of the phenolic proton at δ = 10.70 ppm disappears in the complex spectrum. It is replaced by a new broad signal at δ = 12.90 ppm, which may correspond to NH^+^, indicating the forming of a proton transfer complex consistent with FTIR results [12,44]. Moreover, the complex spectrum shows signals at δ = 5.45 and 7.27 ppm, which are assigned to the protons of two amino groups. These signals appear downfield relative to those of free 3,4-DAP, which are observed at 4.50 and 5.33 ppm. Additionally, as seen in Figures S7 and S8, signals at δ = 6.74, 7.59, and 7.74 ppm, corresponding to the three aromatic protons of the pyridine ring, are shifted compared with their positions in free 3,4-DAP (6.41, 7.49, and 7.66 ppm). A singlet at δ = 7.95 ppm is also observed, which is likely attributable to the symmetrical two protons of DCNP in the PT complex, compared with the signal at 8.27 ppm for free DCNP [44].

The ^13^C NMR spectra provide further proof of the formation of the PT complex (Figures S9–S11). The carbon atoms C5, C2, C4, C6, and C3 are represented by five distinct signals in the spectrum of the free 3,4-DAP at 108.86, 131.18, 135.77, 139.89, and 141.73 ppm, respectively (Figure S10). Most of these signals experience upfield shift through complexation, except for the C3 signal, which shifts downfield to 148.94 ppm compared with its position in the free 3,4-DAP spectrum. This shift suggests a change in the symmetry of 3,4-DAP during complexation (Figure S11). In the case of the symmetrical carbons of DCNP, the C (2,6) and C (3,5) atoms exhibit resonance signals at 122.50 and 124.91 ppm, respectively, in the spectrum of the free proton donor. These signals experience slight upfield shifts to 121.41 and 123.43 ppm upon complexation. Furthermore, the complex exhibits a resonance signal of C1 at 167.58 ppm, while the free DCNP shows a resonance signal of 156.06 ppm. This change indicates the formation of a hydrogen bond between the protonated 3,4-DAP and the deprotonated DCNP molecule, confirming the proton transfer complex. Therefore, there is good evidence for proton transfer and hydrogen bonding in the formed complex from ^1^H and ^13^C NMR, which agrees with FTIR results.

#### 2.2.4. XRD Study

**Figure 6:**
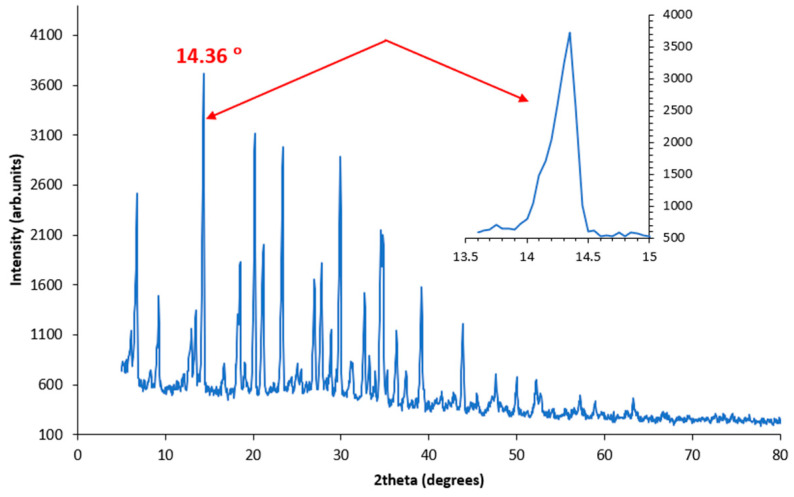
Powder XRD pattern for the PT complex.

The solid PT complex was analyzed using powder X-ray diffraction (XRD) to determine the particle sizes and nanocrystalline structures. Figure 6 displays XRD patterns of the new PT complex. The presence of distinct and clearly defined Bragg peaks at specified 2θ angles confirms the production of crystalline particles in the PT complex. The particle size for the complex was determined by analyzing the full-width at half maximum (FWHM) of the characteristic XRD peaks and their locations. The average particle diameter of the PT complex was determined to be ~40.0 nm, indicating that the particles fall within the nanoscale range. Powder XRD data for characteristic Bragg peaks of the new PT complex are collected in Table 2.

#### 2.2.5. SEM and EDX Studies

**Figure 7:**
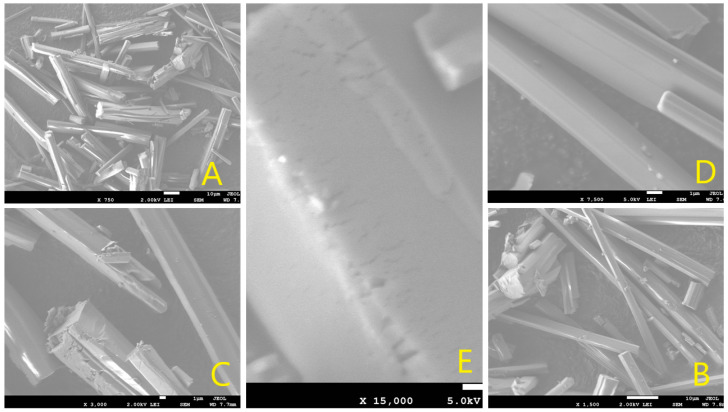
SEM micrographs of the PT complex at various magnification levels (**A** ×750, **B** ×1500, **C** ×3000, **D** ×7500, and **E** ×15,000).

**Figure 8:**
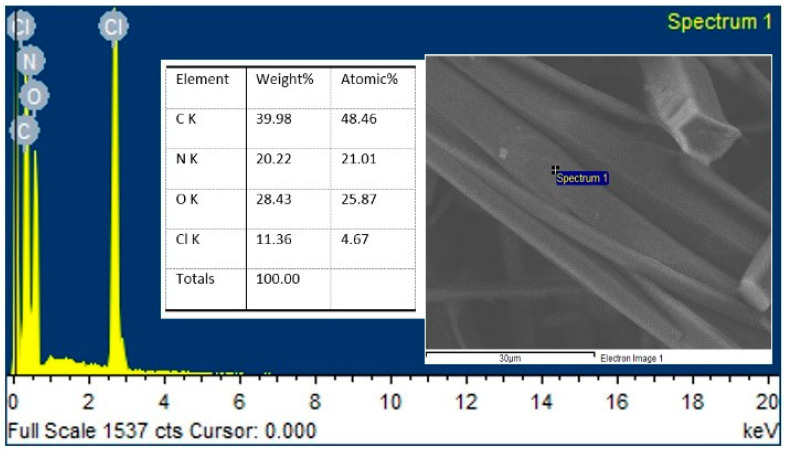
EDX spectrum of the PT complex.

The solid PT complex performed SEM-EDX studies for surface morphology, microstructure, and elemental analysis. Figure 7 displays SEM micrographs obtained at various magnification levels (i.e., ×750, ×1500, ×3000, ×7500, and ×15,000) for the outer surfaces of the complex. The particles of the new complex exhibit apparent shapes, sizes, a homogenously distributed matrix, and a rod-like morphology, as shown in SEM micrographs (Figure 8). A similar morphology was reported for the CT complexes of 3,4-DAP with some e-acceptors [3].

Using the EDX technique, one may obtain information on the chemical composition of a sample, which includes the constituent elements and the distributions and concentrations of those elements. Based on the findings of the EDX analysis, depicted in Figure 8, it was discovered that the newly formed PT complex contained elements such as carbon (C), nitrogen (N), oxygen (O), and chlorine (Cl). This supported the interactions between 3,4-DAP and DCNP to produce the PT complex. As seen in Figure 8, the percentages of elements in the complex are presented as weight percent and atomic percent.

### 2.3. Computational Analysis

#### 2.3.1. Molecular Structure

**Figure 9:**
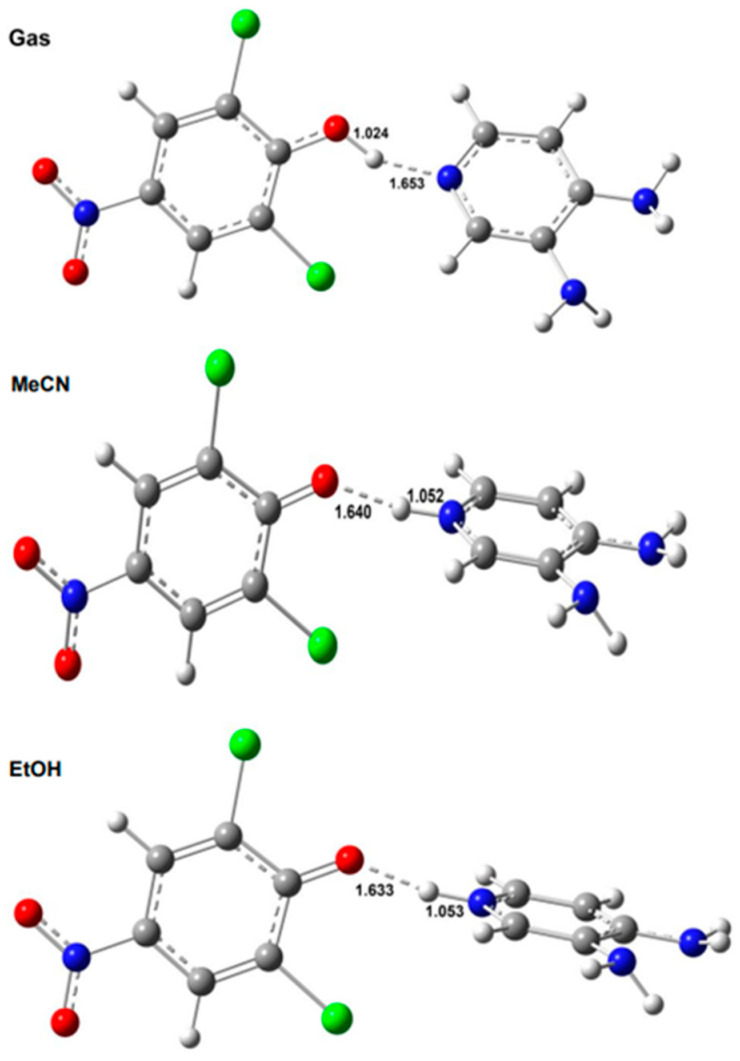
Optimized structures of the PT complex in gas phase, MeCN, and EtOH.

The molecular structure of the studied complex is calculated in the gas phase and the presence of solvent effects (EtOH and MeCN). The optimized structures shown in Figure 9 indicated the presence of significant differences in the proton transfer process depending on the medium. In the gas phase, the O-H distance is calculated to be 1.024 Å while the N…H distance is 1.653 Å, indicating the presence of weak or partial displacement of the O-H proton to the pyridine N-atom. The O-H distance in the case of the free DCNP is 0.971 Å. The results are in good agreement with the dominant electrostatic factor in the gas phase, where the proton favors attaching to the more electronegative O-atom rather than the less electronegative N-atom. In contrast, the presence of solvent–solute interactions in solution has the upper hand over the electrostatic factor in controlling where the proton favors attaching. The solvent effects lead to complete proton transfer from the DCNP to the pyridine N-atom of the 3,4-DAP. The N-H distances are calculated to be 1.052 and 1.053 Å in MeCN and EtOH as solvents, while the O…H distances are 1.640 and 1.633 Å, respectively.

Another geometrical difference was detected between the optimized geometry in the gas phase and those calculated in the presence of solvent effects, which is the degree of twisting between the two hydrogen-bonded moieties. In the gas phase, the two aromatic rings are twisted from each other to a small extent of 39.27° compared with the structure in EtOH and MeCN. In these solvents, the two aromatic ring systems are found almost perpendicular to one another, where the twist angles are calculated to be 86.14 and 79.72°, respectively.

#### 2.3.2. MEP Map and Charge Analysis

**Figure 10:**
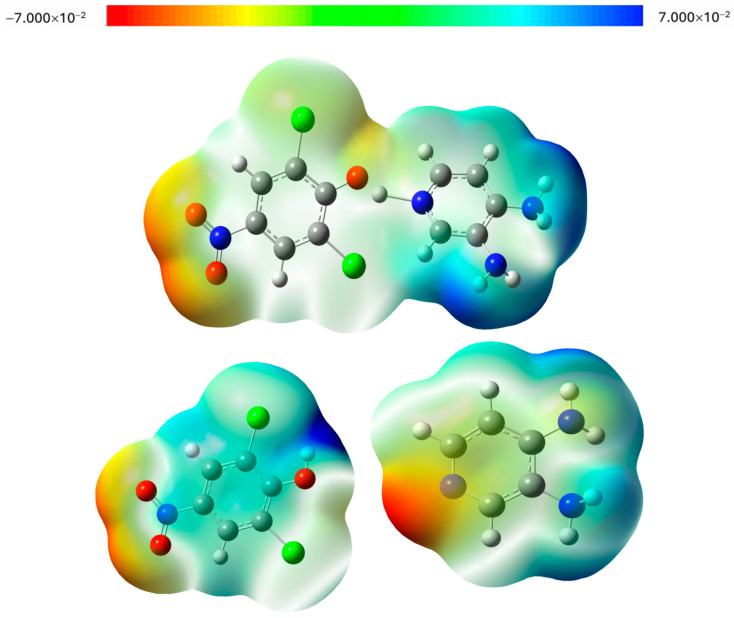
MEP maps for 3,4-DAP and DCNP and their 1:1 complex in the gas phase.

The molecular electrostatic potential maps for the free 3,4-DAP and DCNP molecules, in addition to their hydrogen-bonded complex, are shown in Figure 10. The MEP map is a colored presentation of the charged regions at the different atomic sites. The blue-colored areas are related to the most positive sites, while the red-colored ones are the most negative. The presence of a red area close to the pyridine N-atom indicates that this atomic site is the most suitable for acting as a hydrogen acceptor. On the other hand, the blue color close to the hydroxyl proton indicates that this proton is the most suitable for the proton transfer process. In addition, the colored MEP map for the 1:1 complex showed significant variations because of the proton transfer process. The proton transfer process led to the disappearance of the red region close to the pyridine N-atom and the blue-colored area close to the hydroxyl proton.

In addition, an analysis of the charge distribution at the different atoms compared with the free molecules enabled us to estimate the amount of charge transference from the electron donor species to the electron acceptor one. In the gas phase, a hydrogen-bonded complex exists between DCNP and 3,4-DAP without significant proton transfer. The free molecules have zero formal charges, while the net charges at each moiety in the hydrogen-bonded complex are calculated to be −0.084 and +0.084 e, respectively.

For the isolated DCNP and 3,4-DAP molecules, the net charge is typically zero. On the other hand, the charges of these fragments are altered in the resulting complex as a consequence of the hydrogen bonding interactions as well as the proton transfer process. As a result, the charge analysis also enables us to decide which one is acting as an electron donor or acceptor species. In the gas phase, a hydrogen-bonded complex exists between DCNP and 3,4-DAP without significant proton transfer. The net charges at each moiety in the hydrogen-bonded complex are calculated to be −0.084 and +0.084 e, respectively, instead of zero for the free molecules. Hence, the DCNP is the electron acceptor molecule, while the 3,4-DAP is the electron donor. The results are in agreement with the chemical sense, where the presence of electron-withdrawing groups such as Cl and NO_2_ is the main factor in the high electron acceptor character of DCNP.

In contrast, the presence of two amino groups with strong electron-releasing character through resonance effect agrees with 3,4-DAP being the electron donor one. In the presence of a solvent effect, the complex could also be described as a hydrogen-bonded complex between the protonated 3,4-DAP and the deprotonated DCNP molecule. Assuming these species are in the isolated state, the formal charges are +1 and −1 e, respectively. In EtOH and MeCN as solvents, the net charges of the proton donor are −0.507 and −0.513 e, respectively. In contrast, the net charges for the proton acceptor molecule are +0.507 and +0.513 e, respectively.

#### 2.3.3. UV-Vis Spectra

**Figure 11:**
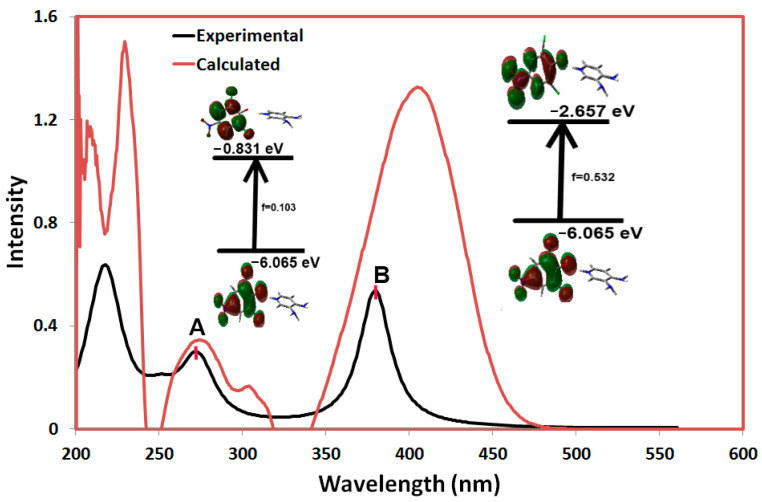
The calculated UV-vis spectrum of the studied complex in EtOH. The excitation bands (**A**) (272.3 nm) and (**B**) (379.5 nm) are observed experimentally at 275 and 406 nm, respectively.

The electronic spectra of the 1:1 PT complex in EtOH are calculated and compared with experimentally determined data in the same solvent. The TD-DFT calculation is used to predict the UV-vis spectra and simulate the experimentally observed one. In addition, the TD-DFT calculation is used to assign the bands observed experimentally. The longest wavelength band observed at 406 nm is calculated at 379.5 nm with oscillator strength (f) of 0.532, which is attributed to HOMO→LUMO transition (99%). As can be seen from Figure 11, the HOMO and LUMO levels are located over the DCNP moiety indicating intramolecular charge transfer-based electronic transition. The shortest wavelength band observed at 275 nm in EtOH is calculated at 272.3 nm (f = 0.103), which is attributed to the HOMO→L+3 transition (87%). This band originated from the intramolecular charge transfer within the deprotonated DCNP moiety, as both HOMO and LUMO+3 are localized over this moiety.

### 2.4. DNA Binding Studies

#### 2.4.1. Electronic Absorption Spectra of Ct-DNA Binding with the PT Complex

**Figure 12:**
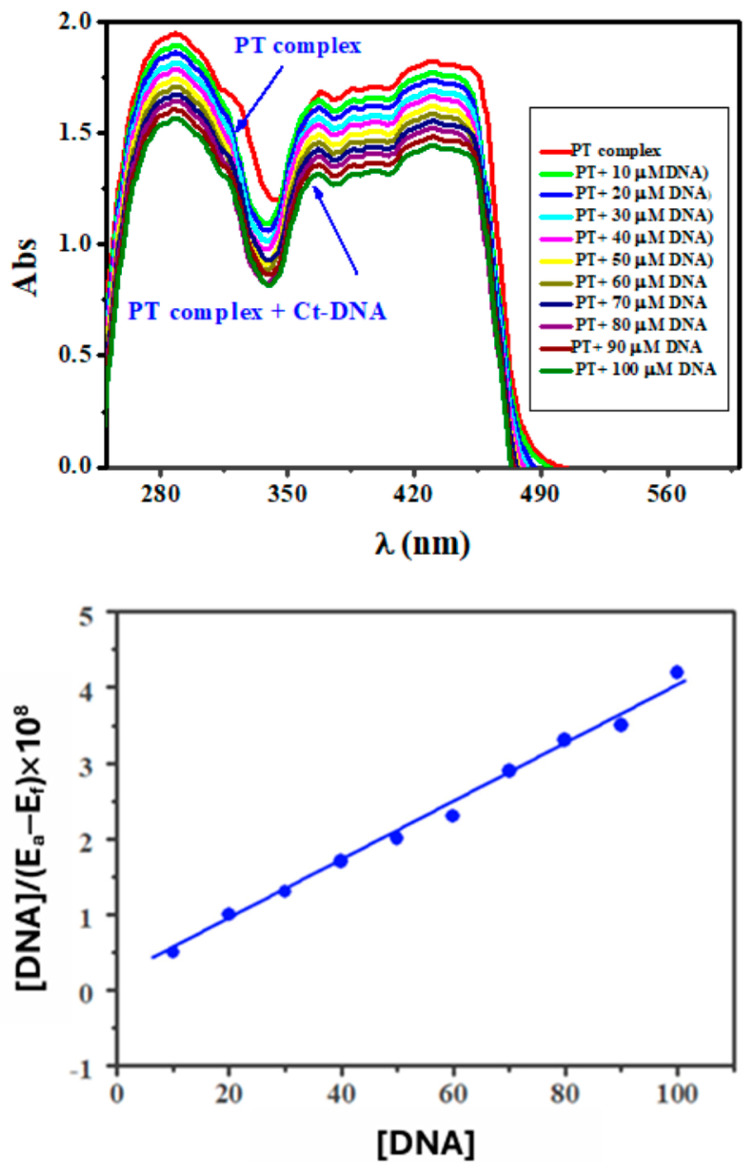
UV-vis absorption spectra of PT complex in Tris buffer (pH 7.5, 298 K) in the absence and presence of Ct-DNA. [PT complex] = 10^3^ μM = 10^−3^ M, [Ct-DNA] = 10–100 μM.

The widely used UV-vis absorption electronic spectroscopy technique is valuable in studying the binding modes of DNA with organic complexes [45,46,47,48]. Hypochromism with or without blue or redshift will result from the binding through intercalation because the aromatic chromophore and the base pair of DNA engage in strong stacking interactions during the intercalative mode [48,49,50,51]. In this work, we compared the absorbance of the PT complex with and without Ct-DNA to investigate the binding mode. Figure 12 displays the absorption spectra of the PT complex with Ct-DNA, which exhibit hypochromism as the concentration of Ct-DNA rises, suggesting the interaction between the PT complex and Ct-DNA. Absorption data were used to determine the intrinsic binding constant (K_b_). Equation (7) can be utilized to ascertain the *K*_b_.

(7) [DNA](εa−εf)=[DNA]1(εb−εf)+1Kb1[εb−εf]

where εf, εb, and εa denote, respectively, the extinction coefficient of the complex when fully bound to DNA, the extinction coefficient at PT complex free in solution, and the extinction coefficient observed for the absorption at a given DNA concentration. *K*_b_ is the ratio of the slope to the intercept on a plot of [DNA]\(εa − εf) versus [DNA] (Figure 12).

The Wolfe–Shimer relationship [52] was used to calculate the intrinsic binding constant (*K*_b_) of the PT complex with Ct-DNA. The PT complex was found to have intrinsic binding constants (*K*_b_) of 4.60 × 10^4^ M^−1^. This value indicates moderate interaction between the PT complex and Ct-DNA, Table 3.

Summarizes the results obtained from applying Equation (8) to determine the free energy (Δ*G*_b_) from the binding constant (*K*_b_) values.

(8) $$∆G_{b}=-RTlnK_{b}$$

#### 2.4.2. Gel Electrophoresis Study

**Figure 13:**
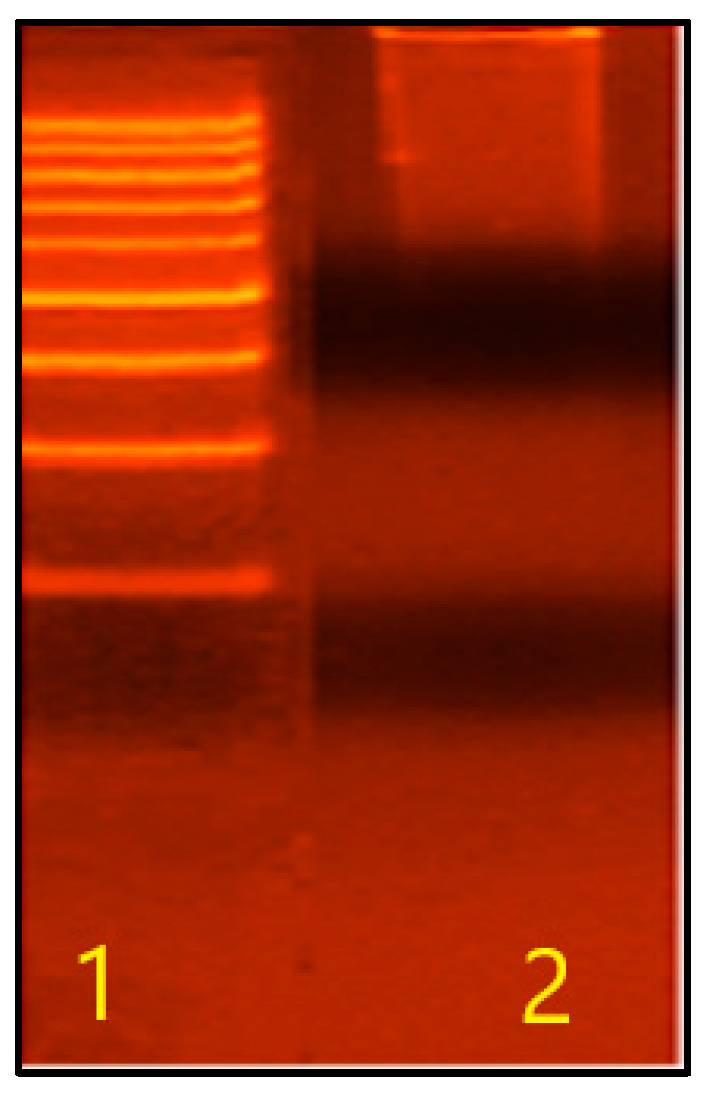
Gel electrophoresis photograph showing the binding interaction of the PT complex with Ct-DNA. Lane 1: DNA ladder, lane 2: PT complex + DNA.

Using agarose gel electrophoresis, the investigation of the interaction between the Ct-DNA and the new PT complexes was facilitated [46,47,48,49,50,51]. The gel electrophoresis picture in Figure 13 demonstrates how the Ct-DNA loaded with the new PT complexes has somewhat decreased in intensity within the PT complex. The experimental findings indicated that an interactive binding mode between Ct-DNA and the new PT complex may occur.

#### 2.4.3. Molecular Docking Study

**Figure 14:**
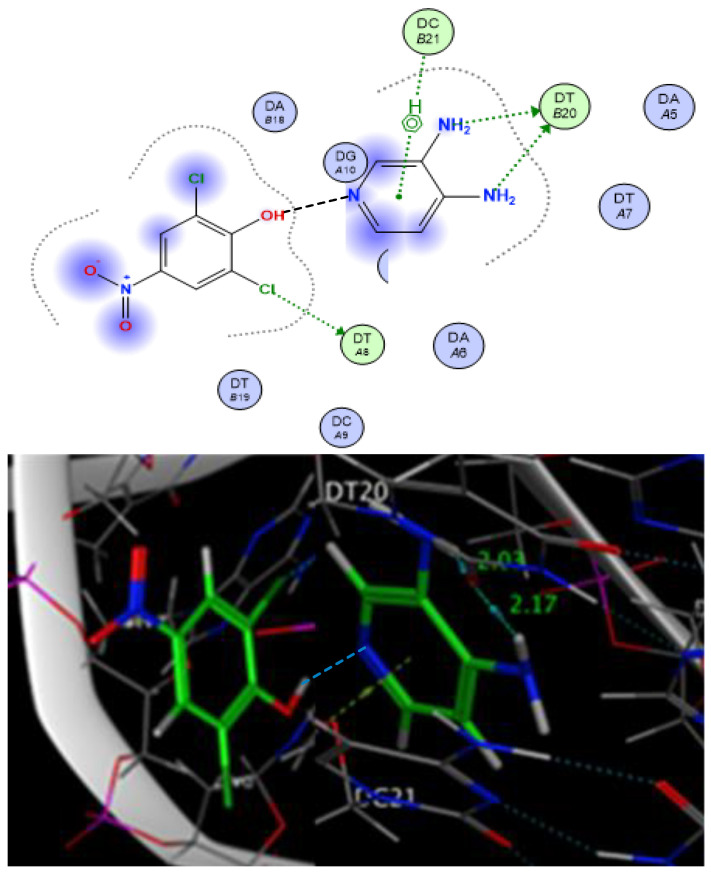
2D and 3D plots of the interaction of the PT complex with the active site of human DNA (PDB ID:1BNA).

The structure of the PT complex was created in a PDB file format from the output of Gaussian09 software. The crystal structures of the receptor of human DNA (PDB ID:1BNA) were downloaded from the protein data bank (https://www.rcsb.org./ accessed on 10 October 2024). The binding free energies of the PT complex with the receptor of human DNA (PDB ID:1BNA), which is a potent mediator of inflammation, are found to be −7.2 kcal/mol, as shown in Figure 14 and Table 4.

### 2.5. Cytotoxicity Activity

**Figure 15:**
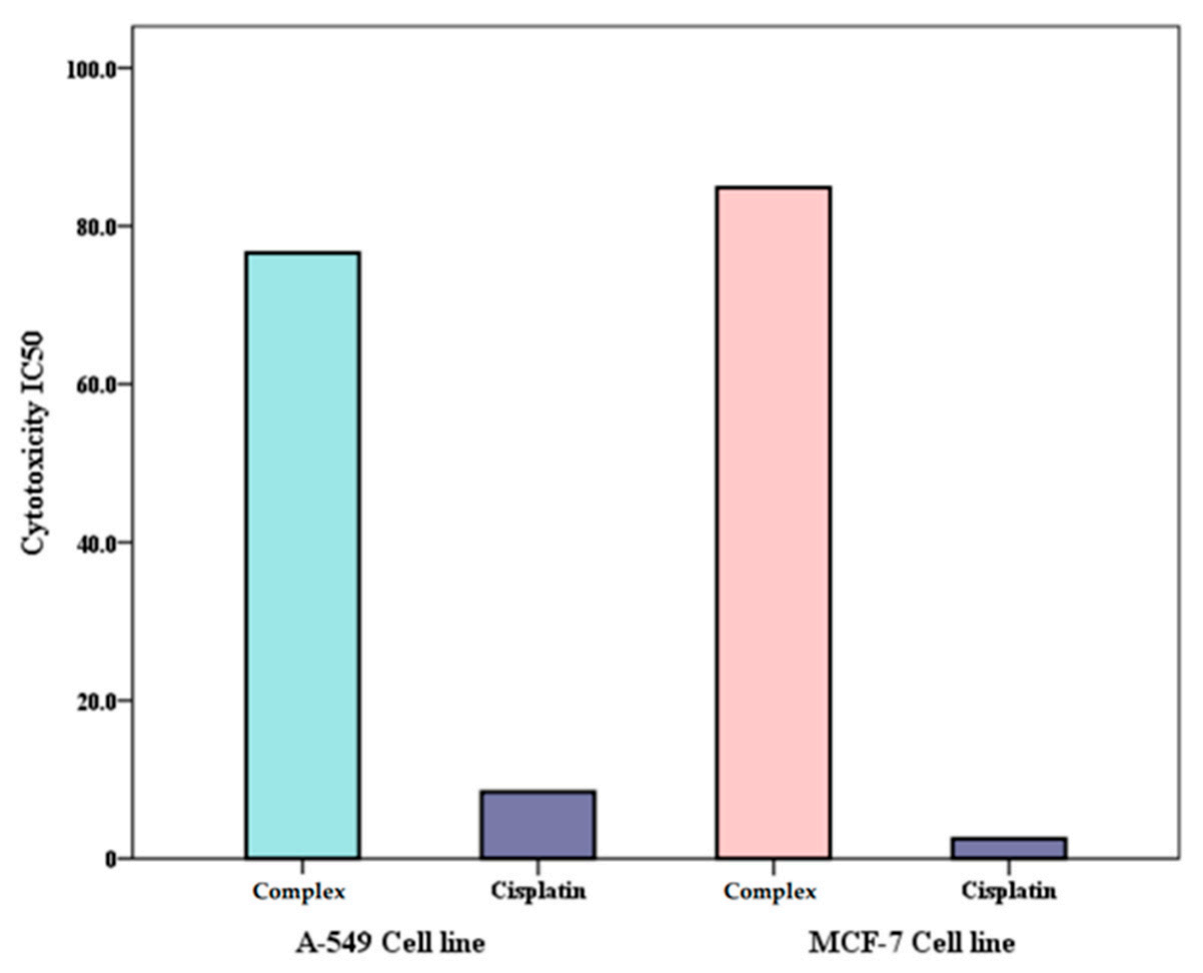
IC_50_ graph of cytotoxicity of the [(3,4-DAP)^+^(DCNP)^−^] complex and cisplatin standard drug that tested for treating A-549 and MCF-7 cancer cell lines.

**Figure 16:**
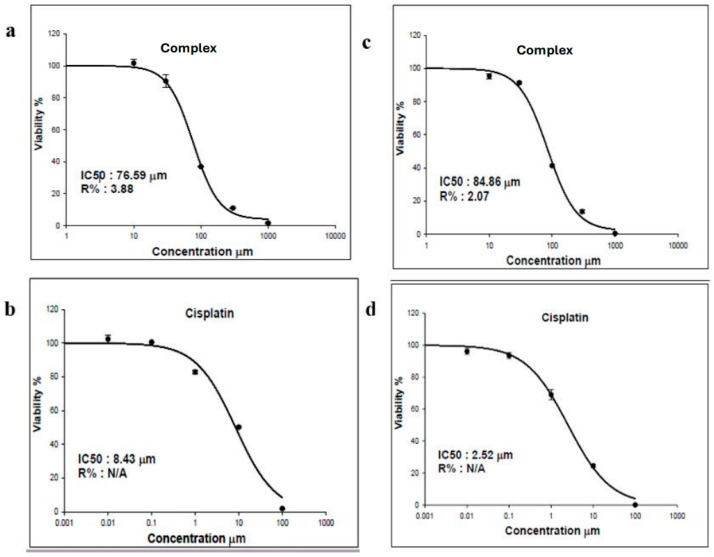
Cell viability curves of A-549 and MCF-7 cancer cell lines that were treated with the PT complex and cisplatin standard drug with various concentrations for 72 h by SRB method. (**a**) A-549 cells treated with the PT complex, (**b**) A-549 cells treated with cisplatin, (**c**) MCF-7 cells treated with the PT complex, and (**d**) MCF-7 cells treated with cisplatin.

The PT complex was further evaluated for its cytotoxicity on human lung cancer A-549 and human breast cancer MCF-7 cell lines using the SRB method. In contrast, cisplatin-treated cells were considered as a positive control; the results are shown in Figure 15 and Figure 16. Different concentrations of the complex (10–1000 μΜ) were performed on the viability of A-549 and MCF-7 cell lines and compared with the drug control of cisplatin in order to study the chemotherapeutic activity of the PT complex as a positive anticancer drug. The IC_50_ graph of cytotoxicity results revealed that the complex inhibited the growth of A-549 and MCF-7 cells in a dose-dependent manner, as shown in Figure 15.

The values (concentration needed for 50% inhibition of cell viability) are calculated using a nonlinear regression of a variable slope model by inserting the various doses applied, the relative cell viability percentage obtained for the PT complex, and the reference drug into the statistical software. The IC_50_ values of PT complex and standard cisplatin against cancer cell lines A-549 and MCF-7 were 76.59 and 8.43 μM, as well as 84.86 and 2.52 μM, respectively (Figure 16). The result demonstrated that the new PT complex showed more cytotoxic effects on A-549 than MCF-7 cells. The cell viability curves of the PT complex treated results on both cell lines treated for 72 h revealed a decline in cell viability.

Notably, these data revealed that the PT complex exhibited more significant cytotoxic effects on A-549 cells compared with MCF-7 cells. Following a 72-h treatment with the PT complex, the viability curves showed a consistent decline in cell viability for both cell lines. However, The anticancer efficacy of PT complex has shown promising results compared with the widely used drug cisplatin. PT complex often exhibits greater cytotoxicity against various cancer cell lines. These complexes typically induce apoptosis through mechanisms involving DNA interaction, reactive oxygen species generation, and cell cycle arrest1. In contrast, although effective, cisplatin is associated with significant side effects and resistance. The ability of PT complexes to overcome cisplatin resistance and reduce side effects makes them a compelling alternative in cancer treatment.

## 3. Materials and Methods

### 3.1. Chemicals

All chemicals were used without further purification and were of analytical grade. DCNP and 3,4-DAP, with a purity level of at least 98%, were purchased from Sigma-Aldrich Co. (St. Louis, MO, USA). The spectroscopic grade solvents ethanol (EtOH) and acetonitrile (MeCN) were purchased from Fisher Scientific (Hampton, NH, USA). The cell culture medium used in the cytotoxicity investigation was supplied from Sigma-Aldrich Co. (St. Louis, MO, USA), which included Dulbecco’s Modified Eagle’s Medium (DMEM), fetal bovine serum (FBS), streptomycin, penicillin, sulphorhodamine-B (SRB), and trichloroacetic acid (TCA).

### 3.2. Synthesis of Solid PT Complex

**Figure 17:**
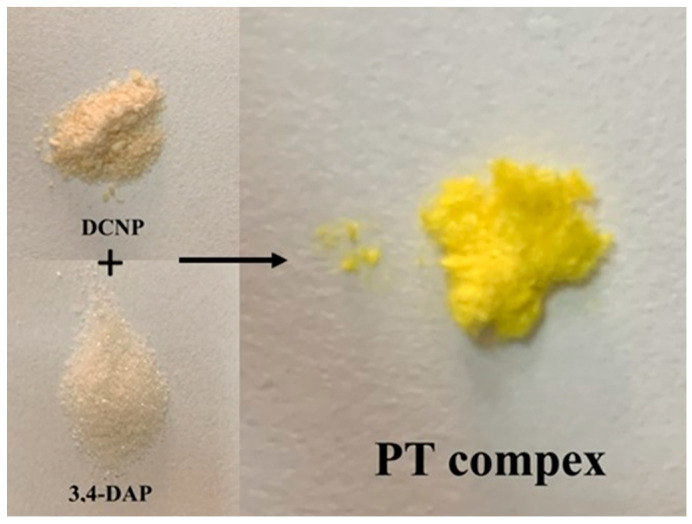
Color of the formed solid product.

Concentrations of 2.0 mmol (0.22 g) of 3,4-DAP and 2.0 mmol (0.42 g) of DCNP were dissolved separately in 25 mL MeCN, then mixed and stirred for about an hour at room temperature (20 °C). A bright yellow product was obtained, filtrated off, washed with a minimum amount of MeCN, and recrystallized from MeCN, then dried in a desiccator (0.523 g, 82.46%) (Figure 17). The stoichiometry of the new product was determined utilizing elemental analysis (CHN). The CHN analysis results were obtained using a Perkin-Elmer 2400 microanalyzer (Waltham, MA, USA). The yellow product melts at 240 °C. Analysis for C_11_H_10_Cl_2_N_4_O_3_, M.wt. 317.13 g mol^−1^, calc.: %C 41.62, %H 3.15, %N 17.65. Found: %C 41.07, %H 3.48, %N 17.47. It is confirmed that the synthesized complex is impurity-free and has a 1:1 ratio by the agreement between the calculated and found CHN results.

FTIR: ν (cm^−1^); 3453 (m), 3409 (w), 3138 (m), 2700 (b) 1679 (m), 1651 (m), 1610 (m), 1559 (s), 1524 (m), 1493 (w), 1467 (m), 1430(m), 1340 (m), 1310 (m), 1293 (m), 1266 (s), 1180 (m), 1140 (s), 896 (s), 853 (w), 830 (w), 798 (s), 752 (s), 715 (m), 651 (m), 626 (m), 571 (m), 497 (m). ^1^H NMR (DMSO-*d*_6_, ppm): δH 12.90 (br s, 1H, NH), 6.74, 7.59, 7.74 (d, 3H, pyridine ring), 5.45, 7.25 (s, 2H, NH_2_), 7.95 (s, 1H, DCNP).; ^13^C NMR (DMSO-*d*_6_, ppm): δC 108.86, 131.18, 135.77, 139.89, 141.73. UV-vis (DMF), λ_max_, nm, (ε, L mol^−1^ cm^−1^), 429 (4952).

### 3.3. Spectroscopy Measurements

The spectroscopic measurements of the new complex were achieved using a Shimadzu UV-Vis 1800 spectrophotometer (Shimadzu, Kyoto, Japan) connected to a Shimadzu TCC-ZUOA temperature controller unit and a personal spectroscopy software version 2.3. The electronic absorption spectra of the molecules were measured in the wavelength range of 700 to 300 nm using a quartz cell with a thickness of 1.0 cm. Stock solutions of donor and acceptor (1.0 × 10^−3^ mol L^−1^) were prepared in EtOH or MeCN and used for further measurements by diluting with solvent. The molecular composition of the PT interaction was established using Job’s method of continuous variations [53], where a series of 9 standard solutions in 10 mL volumetric flasks were prepared, each containing 3,4-DAP and DCNP at varied molar fractions. Each flask’s total solution concentration (3,4-DAP + DCNP) was constant. The absorbance of each solution was measured at the maximum wavelength (λ_max_) against a blank reagent that was handled similarly. The photometric titration also determined the molecular composition using a constant concentration of DCNP (10^−4^ mol L^−1^) with variable concentrations of 3,4-DAP (1.0 × 10^−5^ to 1.4 × 10^−4^ mol L^−1^).

The FT-IR spectra of 3,4-DAP, DCNP, and the formed complex were recorded on a Perkin-Elmer Frontier spectrophotometer (Waltham, MA, USA) as solid-state pellets of KBr in the range of 4000–400 cm^−1^. The ^1^H and ^13^C NMR spectra of reactants and the formed complex were recorded in DMSO-*d*_6_ as solvent and TMS as an internal reference using a Bruker DPX instrument (Billerica, MA, USA) at 700 MHz.

### 3.4. Powder X-Ray Diffraction (XRD) Analysis

Powder X-ray diffraction (XRD) patterns were recorded using a Bruker D8 diffractometer (Billerica, MA, USA) equipped with a copper K-alpha (CuKα) source (wavelength λ = 1.54060 Å). The instrument was operated at 40 kilovolts (kV) and 40 milliamperes (mA). The powdered complex was scanned within the 2θ range of 0° to 80°.

### 3.5. Scanning Electron Microscopy (SEM) Analysis

The SEM-EDX technique was used to examine the surface morphologies of the complex and the elemental compositions. The examination used a Field Emission SEM (Jeol JSM-7600F FESEM, Tokyo, Japan) running at 2.00 kV.

### 3.6. Computational Analysis

Computational analysis was performed using the B3LYP [54,55,56,57] method with 6-31+G(d,p) basis sets with the aid of the Gaussian 09 software [58]. All optimized structures gave no imaginary vibrational modes. GaussView 4.1 [59] was used to draw the MEP and frontier molecular orbitals (FMOs). The origin of the electronic spectra was deduced using the TD-DFT method, which considered the solvent (EtOH) effects using the polarizable continuum model (PCM) [60].

### 3.7. DNA Binding Studies

#### 3.7.1. DNA Binding–Titration Experiments

A stock solution of Ct-DNA was prepared in Tris-HCl buffer (50 mM, pH 7.2) for the DNA binding experiments applying the formed PT complex. Before use, centrifugal dialysis was used to purify the Ct-DNA. The purity of the DNA stock solution was examined by estimating the UV absorbance ratio of the Ct-DNA solution at 260 and 280 nm. The A_260_/A_280_ absorbance ratio was approximately 1.86, suggesting no protein contamination in the Ct-DNA solution [49]. The molar extinction coefficient (e_260_ = 6600 mol^−1^ cm^2^) was applied to record the UV absorbance at 260 nm and calculate the concentration of the Ct-DNA solution per nucleotide. Within a day, the Ct-DNA stock solutions had been utilized after being kept at 4–5 °C. A stock solution of the PT complex with a concentration of 10^3^ μM (10^−3^ M) was prepared by dissolving the appropriate weight of the solid PT complex in DMSO; then, the complex solution was diluted with Tris-HCl buffer for the reaction studies. The Ct-DNA concentrations were varied (10–100 μM), while the PT complex concentration remained constant during the spectrophotometric titration experiments. 

#### 3.7.2. Gel Electrophoresis

A Panasonic Lumix DMC-LZ5K 6MP Digital Camera (Kadoma, Osaka, Japan) was used to capture the PT complex binding gel image with Ct-DNA [45].

### 3.8. Cell Line

Breast Adenocarcinoma (MCF-7) and lung carcinoma (A-549) cell lines were obtained from Nawah Scientific Inc. (Mokatam, Cairo, Egypt). Cells were maintained in DMEM media supplemented with 100 mg/mL of streptomycin, 100 units/mL of penicillin, and 10% of heat-inactivated FBS in a humidified, 5% (*v*/*v*) CO_2_ atmosphere at 37 °C. Cultures containing cells were harvested and tested for viability using an SRB assay.

### 3.9. Cytotoxicity Study

Cytotoxicity was measured using the SRB method [61], using human A-549 and MCF-7 cell lines. Briefly, exponentially growing cells were harvested and suspended in the culture medium (100 mL, DMEM) in a 96-well plate. After 24 h incubation of 100 μL cell suspension (5 × 10^3^ cells) in the complete media at 37 °C under humidified 5% CO_2_, serially-diluted complex solutions (100 mL in DMEM medium) were added to the wells and incubated further for 72 h. The cells were fixed with 150 μL of 10% TCA and incubated at 4 °C for one hour. The TCA solution was removed, and the cells were washed five times with distilled water. This was followed by adding 70 μL SRB solution (0.4% *w*/*v*) and incubating in a dark place at room temperature for 10 min; then, the unbound dye was removed by washing the cells four times with 1% acetic acid and allowed to air-dry overnight. The SRB protein-bound SRB stain was then extracted with 150 μL of TRIS (10 mM) for optical density (OD) determination at 540 nm using a BMG LABTECH^®^-FLUOstar Omega microplate reader (Ortenberg, German).

### 3.10. Molecular Docking Study

The molecular docking investigations were carried out using MOA2022 software [62] to find the possible binding ways of the most active site of the receptor of human DNA (PDB ID:1BNA) [63].

## 4. Conclusions

The 1:1 proton transfer complex between 3,4-DAP and DCNP as hydrogen acceptor and donor, respectively, was synthesized and characterized using elemental analysis, FTIR, UV-Vis, ^1^H, and ^13^C NMR spectra. In addition, surface morphology assessments were conducted utilizing XRD and SEM-EDX spectra, revealing smooth rod-like structures and intricate particles at the nanoscale. DFT calculations predicted the structure of the studied complex in the gas phase and in the presence of solvents (EtOH and MeCN). The slight proton transfer from DCNP to 3,4-DAP in the gas phase could be attributed to the electrostatic factor. In contrast, the complete proton transfer is encouraged in solution due to solute-solvent interactions. The electronic absorption spectra indicate that a proton transfer from DCNP to 3,4-DAP resulted in the production of the phenolate ion. The experimentally observed bands at 406 nm and 275 nm are calculated using TD-DFT calculations at 379.5 nm (HOMO→LUMO) and 272 nm (HOMO→L+3), where both bands belong to intramolecular charge transfer-based electronic transition within the DCNP moiety. The cytotoxic effects of the new complex on A-549 and MCF-7 cancer cell lines were found to be weaker than cisplatin as positive control. The new complex binding with Ct-DNA was studied using UV-Vis absorption spectroscopy and gel electrophoresis. The binding constant indicates a moderate interaction between the PT complex and Ct-DNA through an interactive mode. Changing the substituent on the pyridine ring significantly impacts the anticancer properties of the resulting PT complex. In this regard, other substituted pyridine will be considered in our future work to prepare other PT complexes with improved anticancer characteristics.

## Figures and Tables

**Table 1:** Spectroscopic physical of PT complex at room temperature.

Parameter	Solvent	
	EtOH	MeCN
λ_max_ (nm)	406.0	423.0
*K_f_* (L mol^−1^)	3333	1600
ε_max_	5.0 × 10^4^	12.5 × 10^4^
−ΔG° (kJ mol^−1^)	20.11	18.29
*E*_CT_ (eV)	2.940	3.063
*f*	19.08	34.94
μ (Debye)	46.48	62.90

**Table 2:** Powder XRD data for characteristic Bragg peaks of the PT complex.

2θ (°)	*d* Value (*A*)	FWHM	Intensity (%)	Particle Size (nm)	Mean Particles Size (nm)
6.735	13.11	0.1630	75.99	51.00	40.40
14.36	6.162	0.2250	100.0	37.20	
20.14	4.406	0.1540	81.66	54.70	
21.13	4.201	0.2710	55.00	31.10	
23.35	3.807	0.2310	80.90	36.70	
27.67	3.210	0.2560	49.71	33.40	
29.88	2.987	0.2110	84.88	40.80	
34.57	2.593	0.2180	54.06	39.90	
39.15	2.299	0.2300	48.20	38.90	

**Table 3:** Spectral parameters for the interaction of Ct-DNA with the new PT complex.

λ_max_ Free (nm)	λ_max_ Bound (nm)	∆*n*	Chromism (%) ^a^	Type of Chromism	K_b_ (M^−1^)	∆G	Ε	Assignment
					×10^4^	K J mol^−1^	(dm^3^ mol^−1^ mm^−1^)	
288	289	1	3				1950	π→π*
368	367	1	2				1680	*n*→π*
383	387	4	3	Hypo	4.6	−26.6	1700	*n*→π*
401	400	1	3				1710	Intra ligand band
431	430	1	6				1830	LMCT band
450							1800	d-d band

Chromism%a=Absfree−AbsboundAbsfree.

**Table 4:** Docking interaction data calculations of the PT complex with the active sites of the receptor of human DNA (PDB ID:1BNA).

	Receptor	Interaction	Distance(Å) *	E (kcal/mol)
N 7	O2 DT 20 (B)	H-donor	3.18 (2.03)	−2.5
N 8	O2 DT 20 (B)	H-donor	3.03 (2.17)	−2.7
Cl 7	O2 DT 8 (A)	H-donor	3.55	−1.2
6-ring	C4′ DC 21 (B)	pi-H	3.54	−0.8

* The lengths of H-bonds are in brackets.

## Data Availability

Data are contained within the article and Supplementary Materials.

## Supplementary Materials

The following supporting information can be downloaded at: https://www.mdpi.com/article/10.3390/molecules29215120/s1, Figure S1: Naked-eye visible color change in the solution when adding DCNP (1.0 × 10^−4^ mol L^−1^) to 3,4-DAP (1.0 × 10^−4^ mol L^−1^) in EtOH; Figure S2: Effect of time on the stability of PT complex in different solvents; Figure S3: Electronic absorption spectra of PT complex at different concentrations of 3,4-DAP with 1.0 × 10^−4^ mol L^−1^ in EtOH at room temperature; Figure S4: Electronic absorption spectra of PT complex at different concentrations of 3,4-DAP with 1.0 × 10^−4^ mol L^−1^ in MeCN at room temperature; Figure S5: Electronic absorption spectra of the solid PT complex in DMF; Figure S6: ^1^H NMR spectrum of the free DCNP; Figure S7: ^1^H NMR spectrum of the free 3,4-DAP; Figure S8: ^1^H NMR spectrum of PT complex; Figure S9: ^13^C NMR spectrum of the free DCNP; Figure S10: ^13^C NMR spectrum of the free 3,4-DAP. Figure S11. ^13^C NMR spectrum of PT complex.
