# Supplementary material for: A New Proton Transfer Complex Between 3,4-Diaminopyridine Drug and 2,6-Dichloro-4-nitrophenol: Synthesis, Spectroscopic Characterization, DFT Studies, DNA Binding Analysis, and Antitumor Activity
Source: Molecules. 2024 Oct 30;29(21):5120. doi: 10.3390/molecules29215120 (PMC11547504; doi:10.3390/molecules29215120)
Supplement: Supplementary file 1 [file molecules-29-05120-s001.zip › molecules-3240610-supplementary.pdf]

A new proton transfer complex between 3,4-diaminopyridine drug and 2,6-dichloro-4-nitrophenol: Synthesis, spectroscopic characterization, DFT studies, DNA binding analysis, and antitumor activity.

Reem M. Alghanmi [1,*], Maram T. Basha [1], Ahlam I. Al-Sulami [1], Saied M. Soliman [2], and Laila H. Abdel-Rahman [3]

[1] Department of Chemistry, College of Science, University of Jeddah, P.O. 80327, Jeddah 21589, Saudi Arabia; mtbasha@uj.edu.sa (M.T.B.); aialsulami@uj.edu.sa (A.I.A.).
[2] Department of Chemistry, Faculty of Science, Alexandria University, P.O. 426, Ibrahimia, Alexandria 21525, Egypt; saied1soliman@yahoo.com
[3] Chemistry Department, Faculty of Science, Sohag University, 82534 Sohag, Egypt; laila.abdelrahman@science.sohag.edu.eg
* Correspondence: rmalghanmi@uj.edu.sa

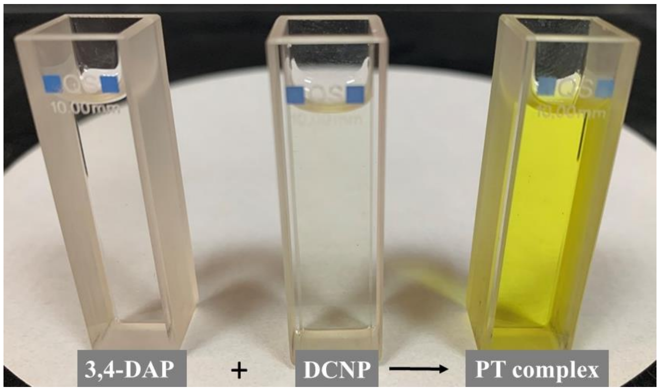

Figure S1. Naked-eye visible color change in the solution when adding DCNP (1.0×10^{-4} mol L^{-1}) to 3,4-DAP (1.0×10^{-4} mol L^{-1}) in EtOH.

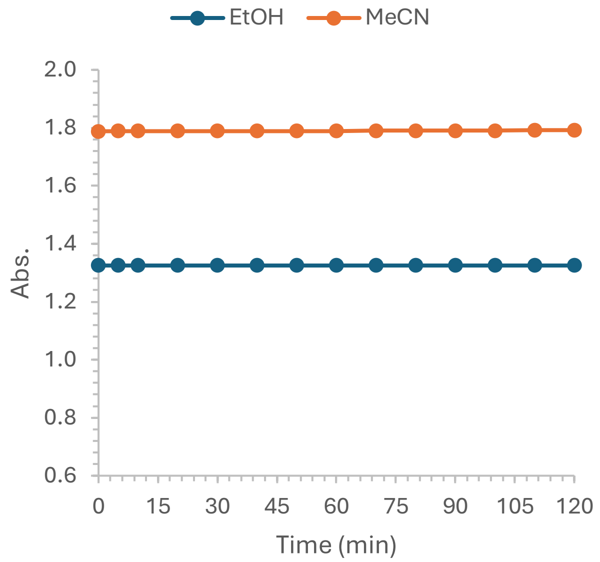

Figure S2. Effect of time on the stability of PT complex in different solvents.

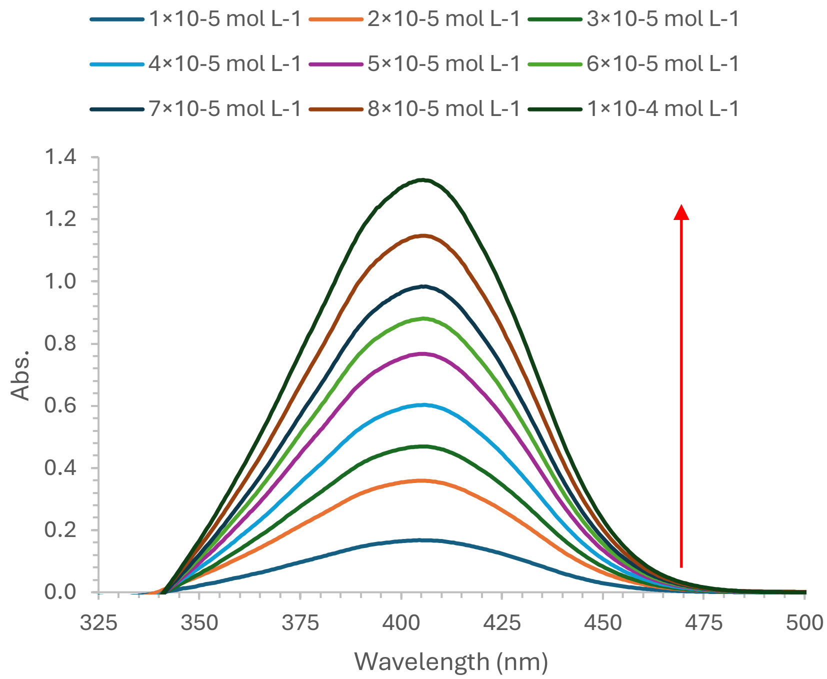

Figure S3. Electronic absorption spectra of PT complex at different concentrations of 3,4-DAP with 1.0 × 10^{-4} mol L^{-1} in EtOH at room temperature.

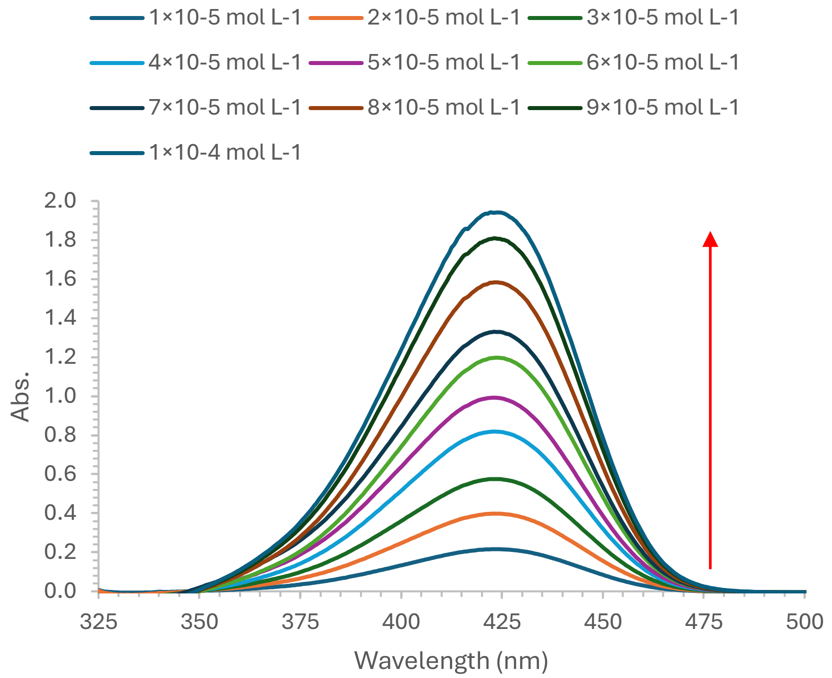

Figure S4. Electronic absorption spectra of PT complex at different concentrations of 3,4-DAP with 1.0 × 10^{-4} mol L $^{-1}$ in MeCN at room temperature.

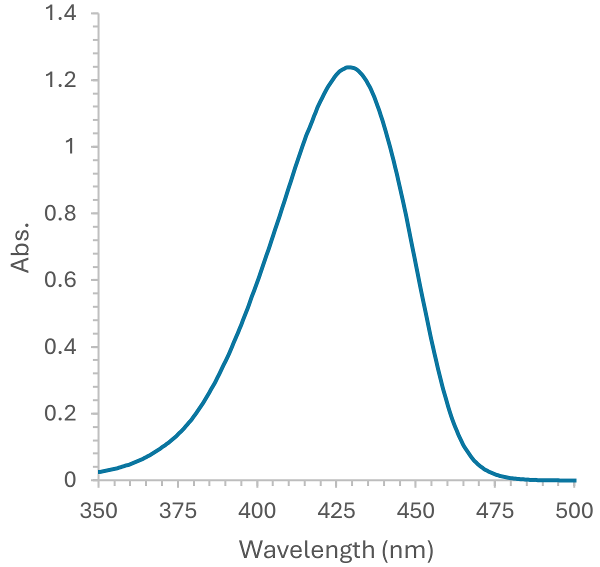

Figure S5. Electronic absorption spectra of the solid PT complex in DMF.

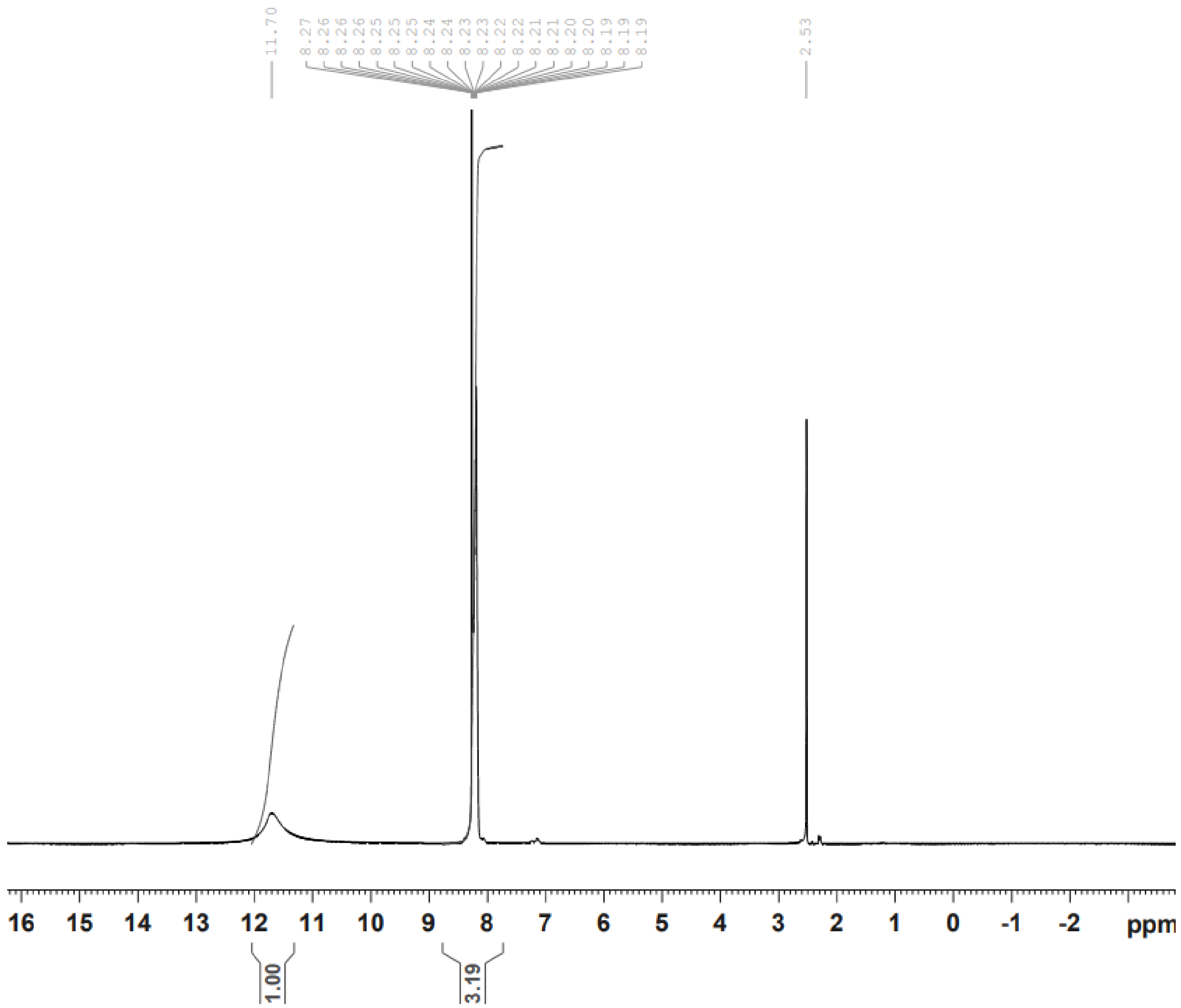

Figure S6. ^{1}H NMR spectrum of the free DCNP.

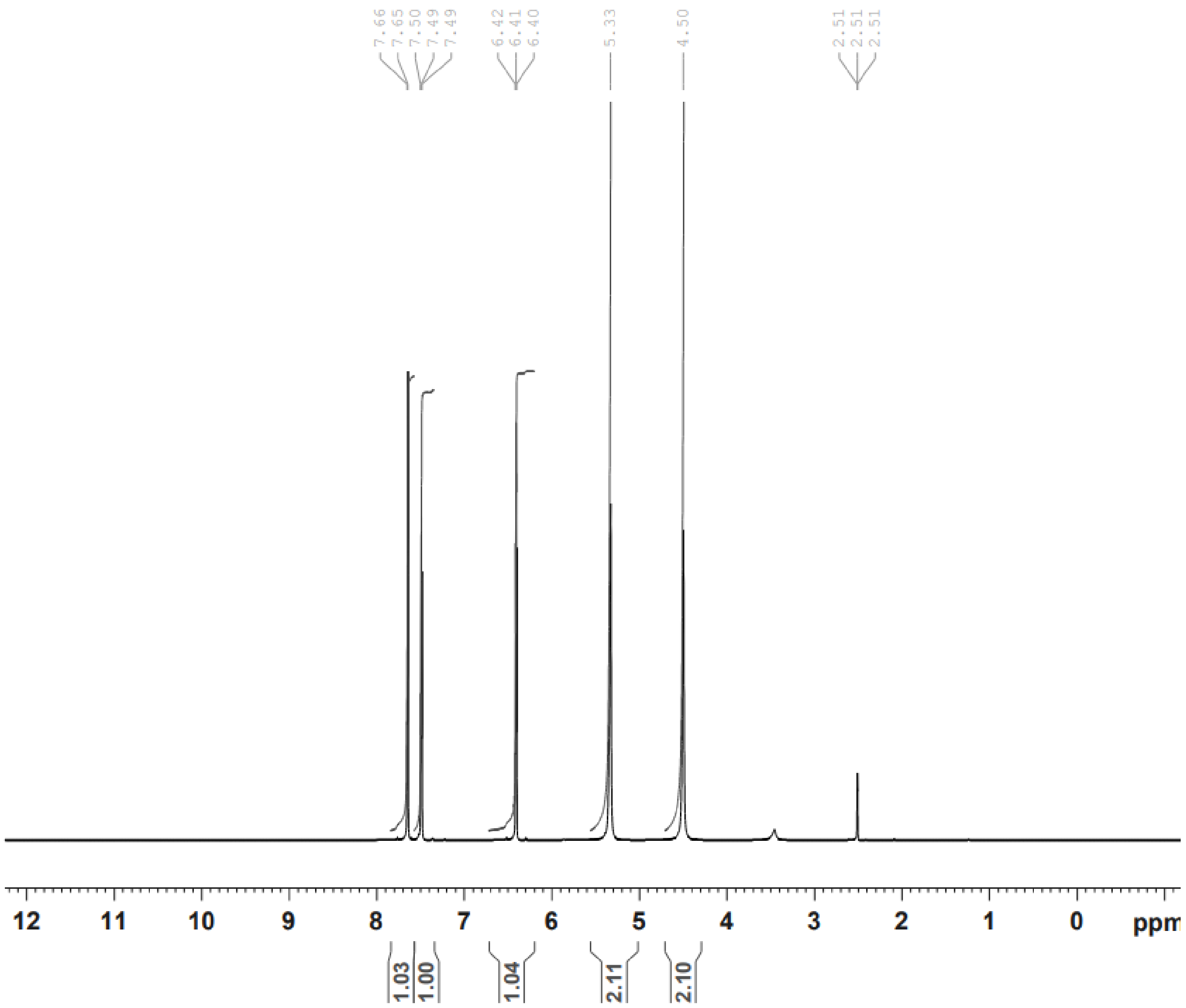

Figure S7.^{1}H NMR spectrum of the free 3,4-DAP.

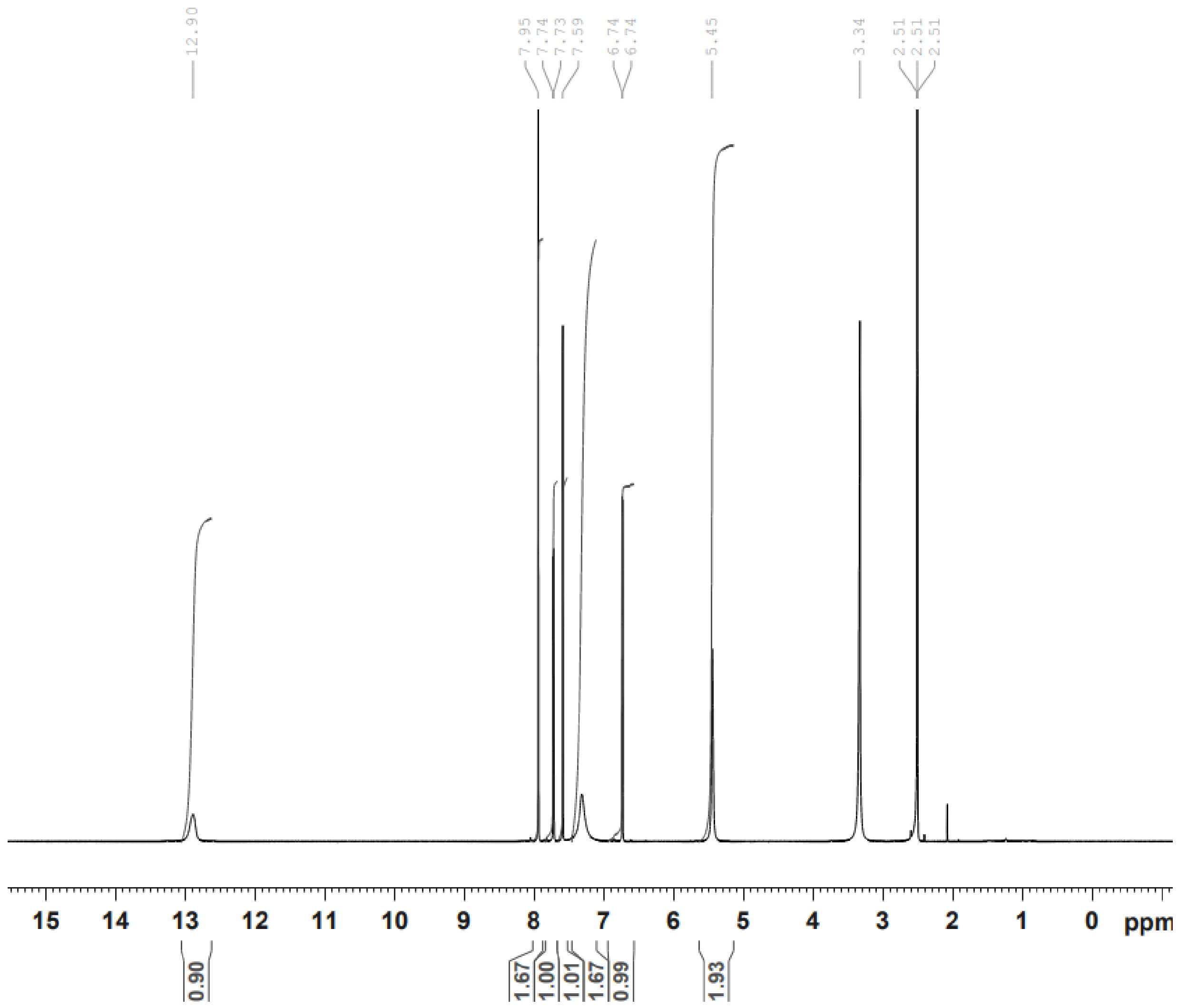

Figure S8. ^{1}H NMR spectrum of PT complex.

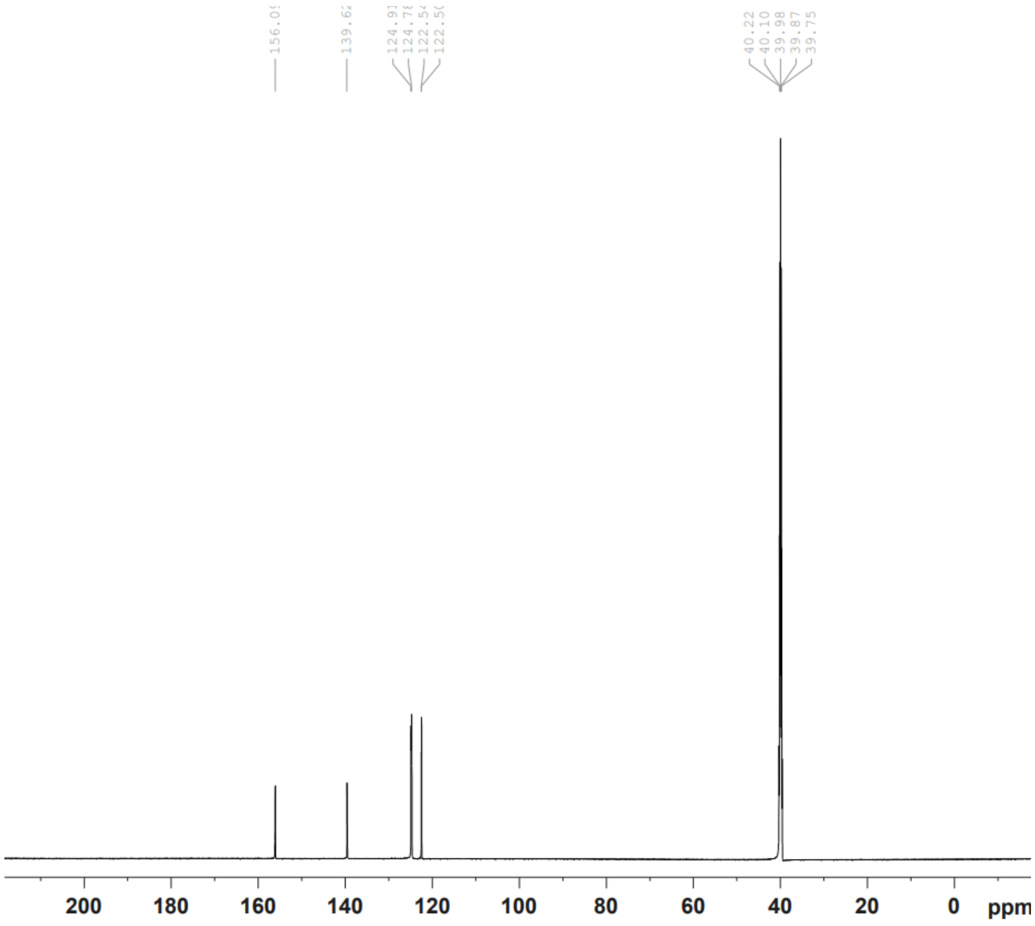

Figure S9. ^{13}C NMR spectrum of the free DCNP.

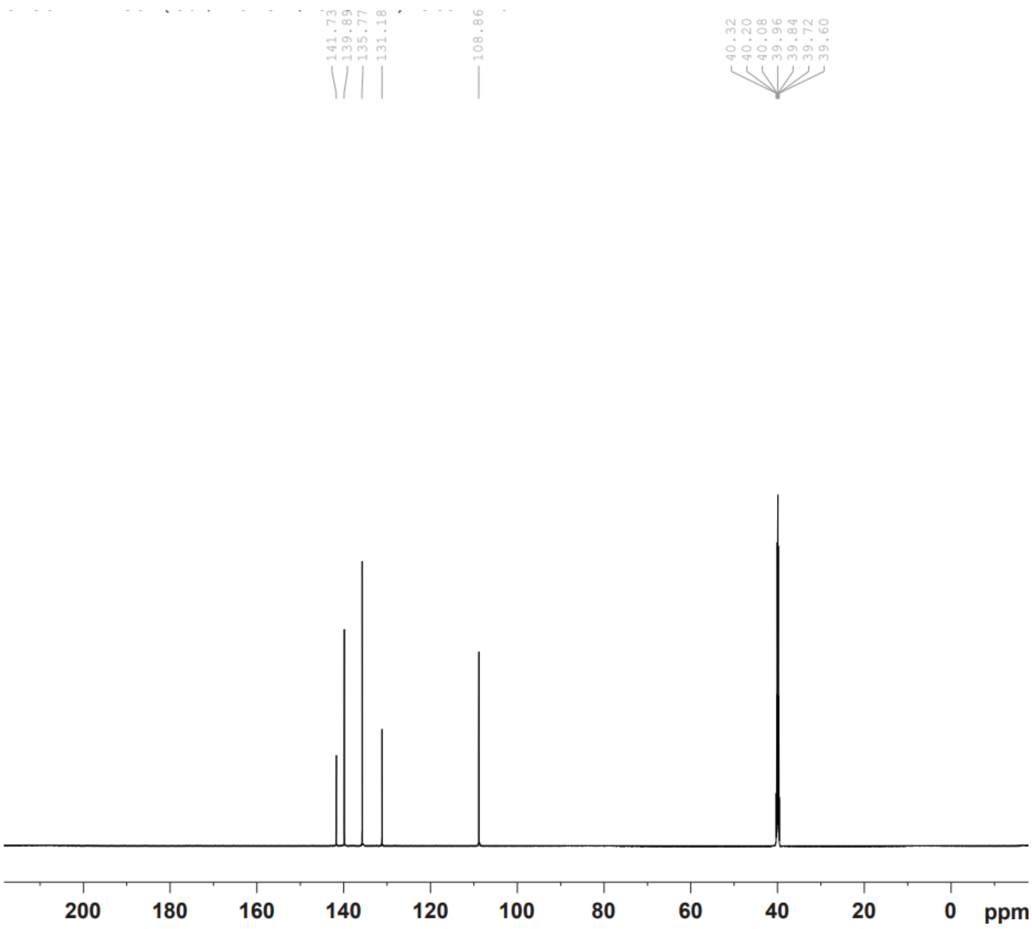

Figure S10. ^{13}C NMR spectrum of the free 3,4-DAP.

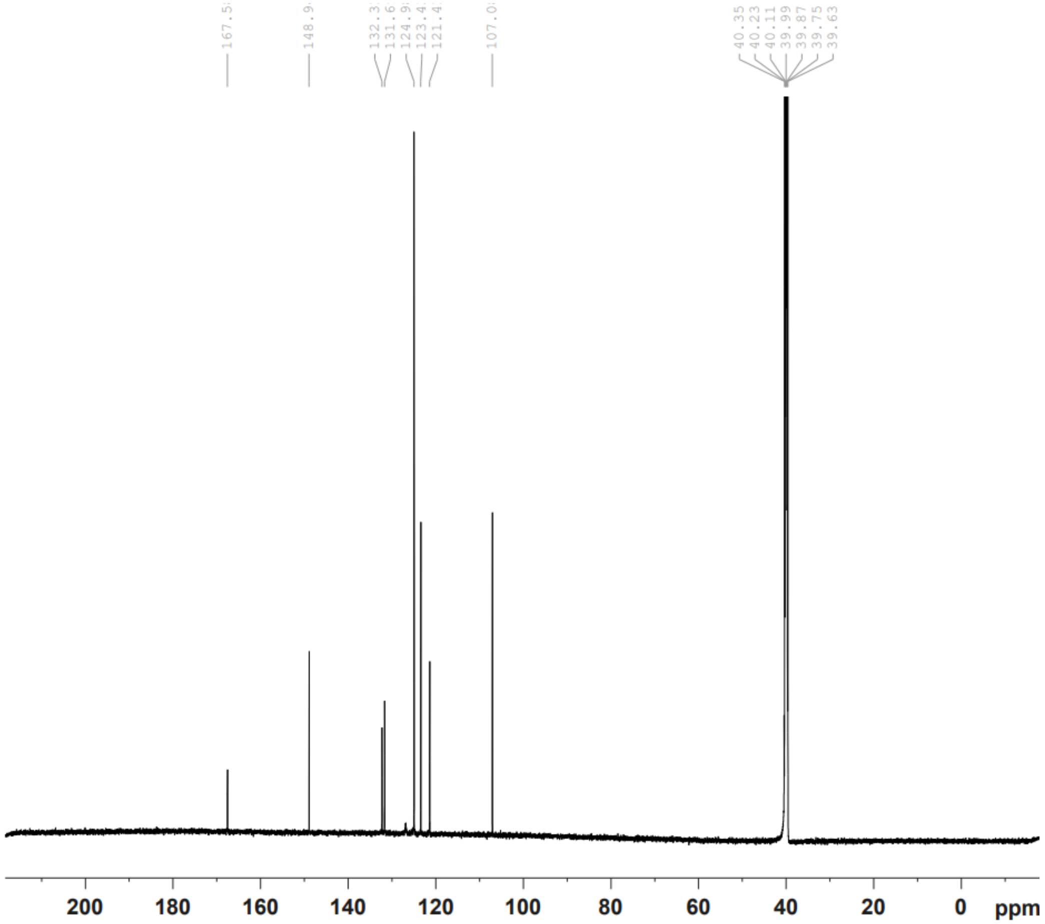

Figure S11. ^{13}C NMR spectrum of PT complex.
